# Oceanapiside, a Marine Natural Product, Targets the Sphingolipid Pathway of Fluconazole-Resistant *Candida glabrata*

**DOI:** 10.3390/md19030126

**Published:** 2021-02-26

**Authors:** Doralyn S. Dalisay, Evan W. Rogers, Tadeusz F. Molinski

**Affiliations:** 1 Department of Chemistry and Biochemistry, University of California, San Diego, 9500 Gilman Drive, La Jolla, CA 92093, USA; ddalisay@usa.edu.ph (D.S.D.); rogers_evan@hotmail.com (E.W.R.); 2 Center for Chemical Biology and Biotechnology (C2B2) and Department of Biology, College of Liberal Arts, Sciences and Education, University of San Agustin, Iloilo City 5000, Philippines; 3 Skaggs School of Pharmacy and Pharmaceutical Sciences, University of California, San Diego, 9500 Gilman Drive, La Jolla, CA 92093, USA

**Keywords:** antifungal, Porifera, azole, long-chain base, sphingolipid

## Abstract

Oceanapiside (OPS), a marine natural product with a novel bifunctional sphingolipid structure, is fungicidal against fluconazole-resistant *Candida glabrata* at 10 μg/mL (15.4 μM). The fungicidal effect was observed at 3 to 4 h after exposure to cells. Cytological and morphological studies revealed that OPS affects the budding patterns of treated yeast cells with a significant increase in the number of cells with single small buds. In addition, this budding morphology was found to be sensitive in the presence of OPS. Moreover, the number of cells with single medium-sized buds and cells with single large buds were decreased significantly, indicating that fewer cells were transformed to these budding patterns, suggestive of inhibition of polarized growth. OPS was also observed to disrupt the organized actin assembly in *C. glabrata*, which correlates with inhibition of budding and polarized growth. It was also demonstrated that phytosphingosine (PHS) reversed the antifungal activity of oceanapiside. We quantified the amount of long chain-bases (LCBs) and phytoceramide from the crude extracts of treated cells using LC-ESI-MS. PHS concentration was elevated in extracts of cells treated with OPS when compared with cells treated with miconazole and amphotericin B. Elevated levels of PHS in OPS-treated cells confirms that OPS affects the pathway at a step downstream of PHS synthesis. These results also demonstrated that OPS has a mechanism of action different to those of miconazole and amphotericin B and interdicts fungal sphingolipid metabolism by specifically inhibiting the step converting PHS to phytoceramide.

## 1. Introduction

The widespread and extensive use of antifungal fluconazole (FLC) for the treatment of *Candida* infections has led to increased clinical emergence of fluconazole-resistant, non-*Candida albicans Candida* species, primarily *Candida glabrata* [1,2,3,4]. In fact, *C. glabrata* is frequently isolated from patients receiving fluconazole (FLC) treatment [2,5,6,7,8,9] and consistently considered as the second most persistent fungal pathogen in the United States, mainly causing systemic and mucosal infections in immunocompromised hosts [6,8,10]. The innate fluconazole resistance of *C. glabrata* makes it difficult to manage because of limited number of effective antifungal agents [8,10] and its innate mutations on the overexpression of the drug target (i.e., Erg11) and induction of the drug efflux system (e.g., Pdr1) [11,12]. A member of the “azole” class of antifungals, FLC and commonly prescribed drugs for treatment of candidiasis selectively inhibit ergosterol biosynthesis [13]. The mode of action is fungistatic, requiring maintenance therapy to suppress recurrence of infection. Widespread non-compliant azole usage is often encountered with emergence of fluconazole-resistant strains. *C. glabrata* was also reported to show innate lower susceptibility to amphotericin B (AMB) [10,14], a polyene macrolide that is active in both resting and growing fungal cells. AMB binds selectively to membrane-bound ergosterol, resulting in the formation of membrane pores, followed by cell lysis and death [15]. The substantial toxicity of AMB and low-therapeutic index of AMB and other polyenes limits their usage. The liabilities of both azoles and polyenes highlight the need for new antifungal agents with complementary mechanisms of action (MOAs).

**Figure 1:**
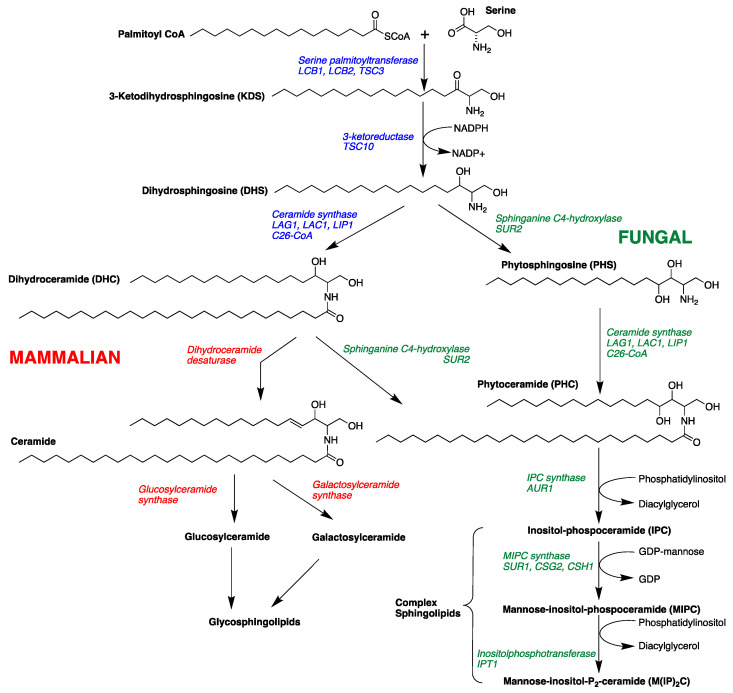
Sphingolipid metabolism in fungal and mammalian cells showing intermediates and enzymes and genes (italics) [30,31].

The sphingolipid biosynthetic pathway has been suggested as a novel target for antifungal therapeutics [16,17,18,19,20,21,22,23]. Sphingolipids are composed of a sphingoid long-chain amino base, generally C_18_, with the 2-amino group acylated by a fatty acid. Fatty acyl sphingosines or ceramides are predominantly found as structural components of plasma membranes [24,25]. Long-chain bases (LCBs), long-chain base phosphates (LCBPs), and ceramides (*N*-acyl-LCBs) also act as signaling molecules, which affect cell growth, endocytosis, cell differentiation, and cell death [24,25,26]. Sphingolipid biosynthesis in mammalian and fungal cells is similar in the early steps of the pathway. Condensation of palmitoyl-CoA and serine gives 3-ketodihydrosphingosine followed by NADPH-dependent reduction to form dihydrosphingosine (DHS, Figure 1) [19,24,25]. Dihydrosphingosine (DHS) undergoes acylation to form dihydroceramide (DHC) by the action of (dihydro) ceramide synthases Lag1, Lac1, and Lip1 [27,28]. Mammalian and fungal sphingolipid biosynthesis diverge at this point. In yeast cells, DHS/DHC undergoes C_4_ hydroxylation through the action of Sur2 [29] to form phytosphingosine (PHS) and phytoceramide (PHC), respectively. In mammalian cells, DHC rapidly dehydrogenated to give ceramide. The next step in fungal biosynthesis is acylation of PHS to yield phytoceramide, a C_26_ fatty acyl substituted metabolite. PHC is incorporated into complex sphingolipids (phosphoinositol containing sphingolipids), which are not found in mammalian cells [30,31]. Thus, critical differences in complex sphingolipid metabolism may be useful points of intervention for antifungal therapy.

**Figure 2:**
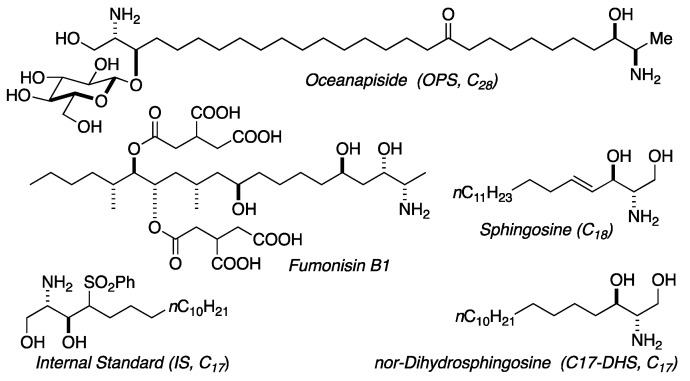
Structures of oceanapiside (OPS), sphingosine, fumonisin B1, and LCMS standards.

We previously reported the isolation and structure elucidation of the antifungal compound, oceanapiside, from the marine sponge *Oceanapia phillipensis* [32,33]. Oceanapiside (OPS), has a novel chemical structure, a glycosylated “two-headed” α,ω-*bis*-amino alcohol with chain termini that resemble the functional head groups of sphinganine (dihydrosphingosine) and fumonisins (e.g. fumonisin B1), respectively (Figure 2). OPS exhibits potent selective activity against fluconazole-resistant *C. glabrata* (Minimum Inhibitory Concentration, MIC 10 μg/mL or 15.4 μM), while oceanalin (the corresponding aglycone obtained by methanolysis of OPS) is significantly more potent (MIC 3 μg/mL or 6.2 μM). Oceanapiside shows no activity against *C. albicans* ATCC 14503, *C. albicans* 96–489 and *Candida krusei* [34]. One striking feature of oceanalin is its ten-fold higher antifungal activity when compared to structurally related compounds including D-sphingosine and sphinganine (MIC 30 μg/mL or 100 μM for D-sphingosine; 99.5 μM sphinganine), which suggests a cooperative synergistic action of two-headed sphingoid base [34]. The mechanism of antifungal activity of oceanapiside had not been elucidated, but its structural relationship with sphingosine and other sphingolipids suggests the possibility that it may interfere with sphingolipid biosynthesis. The sphingolipid biosynthetic pathway has been suggested as a novel target for antifungal therapy because of the key differences in sphingolipid biosynthesis; complex inositides of LCBs are present in fungi, but absent in mammalian cells [16,17,18,20]. These promising features prompted us to investigate the mode of action of OPS using a dual approach: detailed microbiological investigations of fungal pathogen susceptibility, and biochemical analysis that successfully reveal the site of intervention of OPS in fungal sphingolipid metabolism. The latter was achieved by development of quantitative liquid chromatography mass spectrometric (LCMS) assay of key intermediates in sphingolipid metabolism in *C. glabrata*, followed by careful measurement of time-dependent sphingolipid concentrations in the presence of OPS. 

## 2. Results and Discussion

### 2.1. Effect of Oceanapiside (OPS) on Cell Viability in C. glabrata

**Figure 3:**
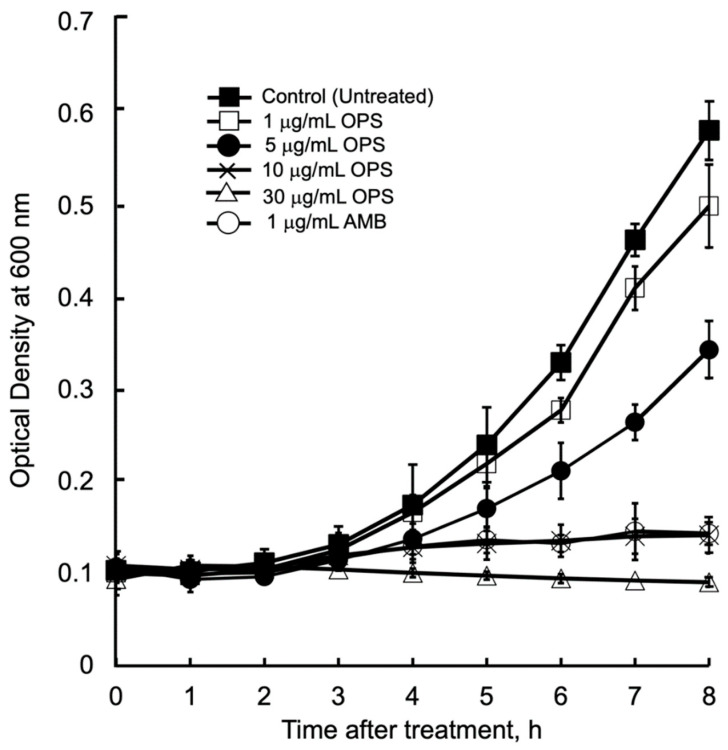
*Candida glabrata* growth inhibition kinetics in the presence of oceanapiside (OPS) at different concentrations. *C. glabrata* cells were resuspended in fresh sabouraud dextrose (SD) medium containing the desired concentration of OPS, amphotericin B (AMB), or 1% DMSO (negative control) and incubated at 37 °C. Growth was measured by optical density (λ = 600 nm). The data represent three separate experiments and are presented as mean values +/− Standard Deviation.

The antibacterial kinetics of OPS at different concentration against *C. glabrata* in liquid media were evaluated by measuring the OD (*λ* = 600 nm) after culturing from 1 to 8 h. (Figure 3). The initial growth of *C. glabrata* was time dependently delayed, along with the increased concentrations of OPS. For *C. glabrata* cells treated with OPS at 1 and 5 μg/mL, delays in growth were observed 3 h after treatment, however, the growth increased with time. It was noticeable that OPS at its MIC 10 μg/mL and MIC 3× 30 μg/mL were strongly inhibitory to *C. glabrata*, showing no growth in the liquid medium until 8 h of treatment. The same trend of growth inhibition was observed for *C. glabrata* incubated with 1 μg/mL AMB. Moreover, the cells treated with 10 and 30 μg/mL OPS were not viable when streaked onto fresh SD agar plate after 8 h of treatment, indicating a fungicidal effect of OPS. Unlike the work of Nicolas et al. [34] that showed fungicidal effect after 18 to 24 h of OPS treatment, our work provides new insight that fungicidal activity of OPS against *C. glabrata* was attained at shorter times (i.e., 8 h) after OPS treatment. Further, in comparison to AMB, which induces immediate cell lysis and death upon exposure, the antifungal activity of OPS was associated with cell growth. OPS was found to be fungicidal against growing *C. glabrata* cells, and the inhibitory activity took effect after 3 to 4 h of exposure.

**Figure 4:**
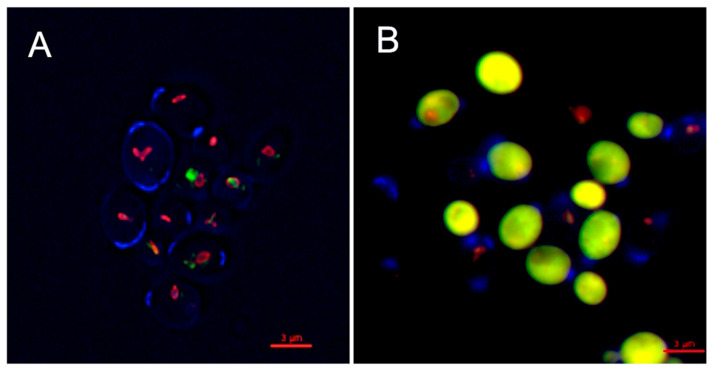
Inhibitory activity of oceanapiside (OPS) against fluconazole-resistant *C. glabrata* cells using LIVE/DEAD yeast viability kit (Molecular Probes, Eugene, OR, USA). An overnight culture of *C. glabrata* was incubated at 37 °C in the presence or absence of 10 µg/mL OPS for 2 h. Yeast cells were stained with FUN1 dye and counterstained with Calcofluor White M2R. The cells were viewed with an Olympus IX70 Deltavision microscope using a 60× 1.4 NA objective and a 100 W Hg lamp. A multi-pass filter set with the following excitation wavelengths were used: λ 360, 490, and 555 nm. Metabolically active yeast cells contain (**A**) red-fluorescent cylindrical intravacuolar structures (CIVS), while dead cells exhibit (**B**) diffuse yellow-green fluorescence.

The inhibitory activity of OPS against *C. glabrata* was evaluated by viability assay using LIVE/DEAD kit (Molecular Probes, Eugene, OR, USA). The assay distinguishes metabolically active cells from dead cells using fluorescent probe, FUN1 [2-chloro-4-(2,3-dihydro-3-methyl-(benzo-1,3-thiazol-2-yl)-methylidene)1-phenylquinolinium-iodide] stain. FUN1 is a nucleic-acid binding dye that was found to give rise to the formation of cylindrical intravacuolar structures (CIVS, red-fluorescence) in metabolically active cells [35], while dead cells exhibited diffuse yellow-green fluorescence. It is clearly shown in Figure 4 that *C. glabrata* cells were killed upon treatment with OPS (Figure 4B) by the appearance of a bright yellow-green fluorescence in OPS-treated samples. This viability assay demonstrated that OPS effectively killed *C. glabrata* within a short time of exposure (2 h). Moreover, none of the treated cells showed any formation of the CIVS, as compared to that of the untreated cells, wherein prominent CIVS were observed (Figure 4A). To note, ATP is required for CIVS formation [35], thus CIVS are not formed in cells with depleted ATP. The bright yellow green-fluorescent *C. glabrata* cells observed in this study indicate that the cells are metabolically inactive and in a state of ATP depletion. This finding suggests that OPS triggers antifungal activity that halts the production of ATP plausibly by the mechanism proposed in Section 2.3 and Section 2.4. Moreover, it showed that the OPS-treated *C. glabrata* cells were not lysed and still intact.

### 2.2. Oceanapiside, OPS Affects Budding, Polarized Growth, and Actin Assembly in C. glabrata

**Figure 5:**
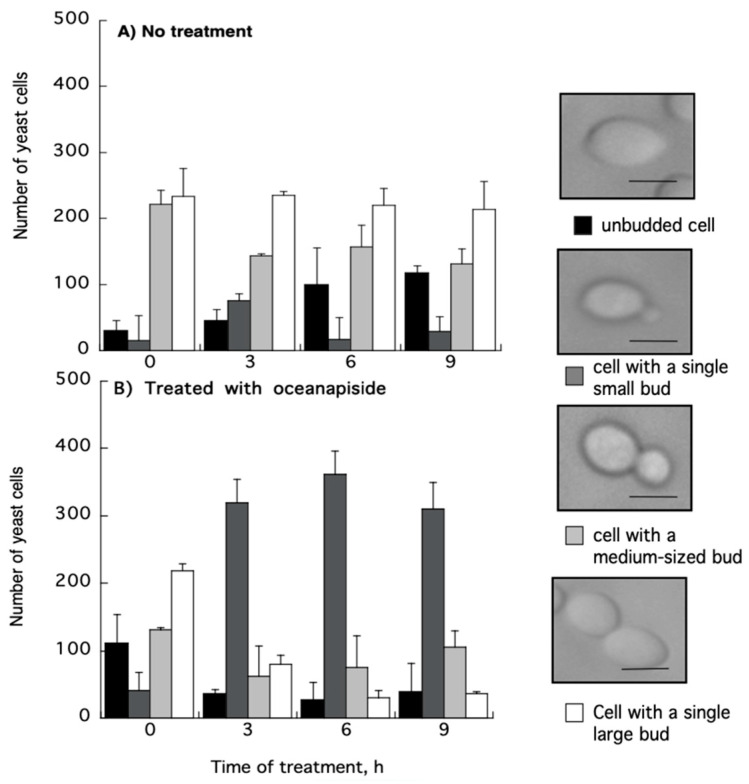
Effect of oceanapiside (OPS) on the proportion of various budding patterns in *C. glabrata*. Cells were treated in the presence or absence of OPS (10 μg/mL) and samples were taken from each culture at 0, 3, 6, and 9 h of incubation. The cells were classified by their budding pattern, as observed under microscope, as follows: unbudded cells; cells with a single small bud; cells with a medium-sized bud; and cells with a large bud. Values are means and standard deviation of the three experiments. Scale bar, 3 μm.

**Figure 6:**
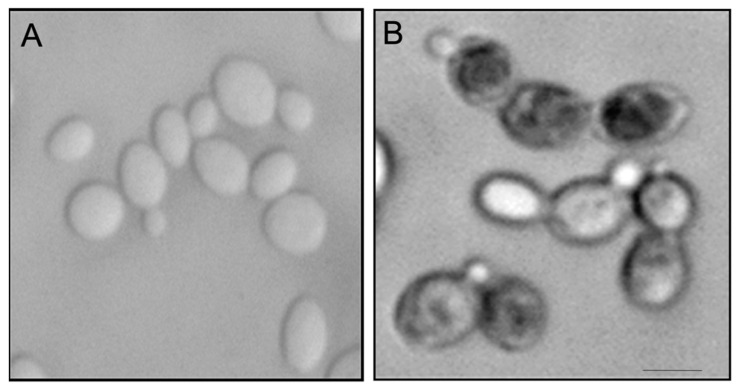
Methylene blue staining of *C. glabrata* in the (**A**) absence or (**B**) presence of OPS (10 μg/mL). An overnight culture of *C. glabrata* was incubated at 37 °C in the presence or absence of OPS (10 μg/mL) for 2 h. The cells were stained with methylene blue (0.1 mg/mL), incubated for 5 min and viewed in a photomicroscope (Nikon). Scale bar, 3 μm.

OPS affected the proportions of *C. glabrata* cells in various budding patterns compared to untreated cells (Figure 5). Although the budding patterns of *C. glabrata* population cells for untreated and OPS treated cells at time 0 h are different due to indiscriminate sampling of cells from SD liquid medium, there was a significant increase in the number of cells with single small buds in the presence of OPS starting from 3 h of incubation and maintained until 9 h. This budding pattern comprises about 70% to 75% of the cell population. It was also observed that the number of cells with single medium-sized buds and cells with single large buds decreased significantly, indicating that fewer cells were transformed to these budding patterns. To know whether these budding patterns were viable or not, the OPS-treated *C. glabrata* cells were stained with methylene blue [36]. Cells with single small buds were deeply stained with methylene blue (Figure 6), indicating that these cells were dead with a collapsed, but not lysed, cell appearance. Cells with medium-sized buds were stained after 9 h of incubation and, in contrast, unbudded cells and cells with single large buds were not stained, suggesting that OPS was less effective against these budding patterns. In contrast, the cells in the untreated culture showed no staining by methylene blue. Collectively, we found that OPS affected the proportion of various budding patterns in *C. glabrata* when compared to untreated cells. The decrease in the number of cells with single medium-sized buds and cells with single large buds indicates that fewer cells are transformed to these budding patterns, which suggest inhibition of polarized growth. Furthermore, the increase in the number of dead cells with small buds suggests that the budding process is halted at this growth stage.

**Figure 7:**
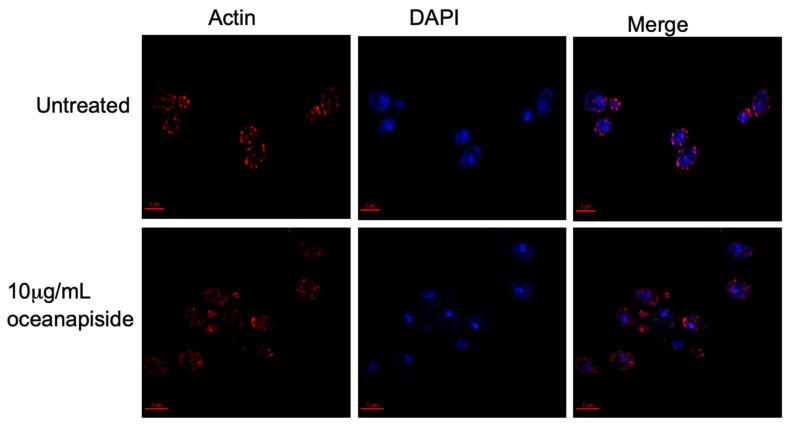
Actin and nucleus staining in fluconazole-resistant *C. glabrata* cells in the presence or absence of OPS 10 μg/mL. An overnight culture of *C. glabrata* was incubated at 37 °C in the presence or absence of 10 μg/mL OPS for 2 h. The cells were fixed; stained with rhodamine-phalloidin, R415 (Molecular Probes, Eugene, OR, USA), to visualize actin; and counterstained with 4′4-diamino-2-phenylindole, DAPI (Sigma), for nucleus staining. The cells were viewed with an Olympus 1X70 Deltavision microscope at 60× 1.4 NA objective and a 100 W Hg lamp. Scale bar, 3 μm.

We demonstrated that OPS affects cellular processes associated with sphingolipid metabolism [24,25,26], specifically on the budding and polarized growth events, as shown by the results above. Sphingoid bases have been reported to mediate signaling cascades in protein phosphorylation, which are directly involved in actin assembly and organization, and are critical for proper budding and polarized growth in yeast cells [37]. It has been reported that inhibition of sphingolipid biosynthesis in *Aspergillus nidulans* rapidly disrupts actin organization, which arrests normal polarized hyphal growth and resulted in the emergence of multiple hyphal branches [38]. Aureobasidin A, an antifungal sphingolipid inhibitor, has been found to disrupt actin assembly and inhibits normal the budding process of *Saccharomyces cerevisiae* [39]. The structural protein, actin, is involved in budding and polarized growth [40,41,42]. In buds, actin is organized in two observable forms, “cables” and “patches”, which correspond to longitudinal growth fibers and bundled actin anchors, respectively. Actin cables and patches participate in determining bud shape and cellular extension [43,44,45,46]. From this context, we hypothesized that OPS affects actin organization and assembly in *C. glabrata* cells. To address this hypothesis, an assay was carried out using fluorescent probes to visualize the actin cytoskeleton organization in OPS-treated *C. glabrata* cells. OPS-treated cells (10 µg/mL) showed numerous red-fluorescent actin patches that were distributed in a disorganized pattern compared to the controls (Figure 7). Moreover, the actin cables, which consist of bundles of actin filaments that generally run along the axis of the budding cells, were not visible in contrast to the untreated cells. This result suggests disruption of organized actin bundles that occur in budding cells and correlates with inhibition of budding and polarized growth in *C. glabrata* observed by methylene blue cytological staining and microscopic evaluation, as described above. Since actin organization is known to be regulated by kinase cascades involving long-chain sphingoid bases (dihydrosphingosine and phytosphingosine)-dependent signaling pathways [25,37], inhibition of sphingolipid biosynthesis is consistent with disruption of sphingolipid biosynthesis in *C. glabrata.* An alternate explanation, that OPS directly disrupts microfilament assembly-disassembly by binding to actin, is possible, but less likely in the face of the biochemical evidence we present here which demonstrates that OPS alters cellular levels of key intermediates en route to the biosynthesis of inositylphosphoceramide (IPC).

### 2.3. Phytosphingosine Reverses the Antifungal Activity of Oceanapiside (OPS)

**Figure 8:**
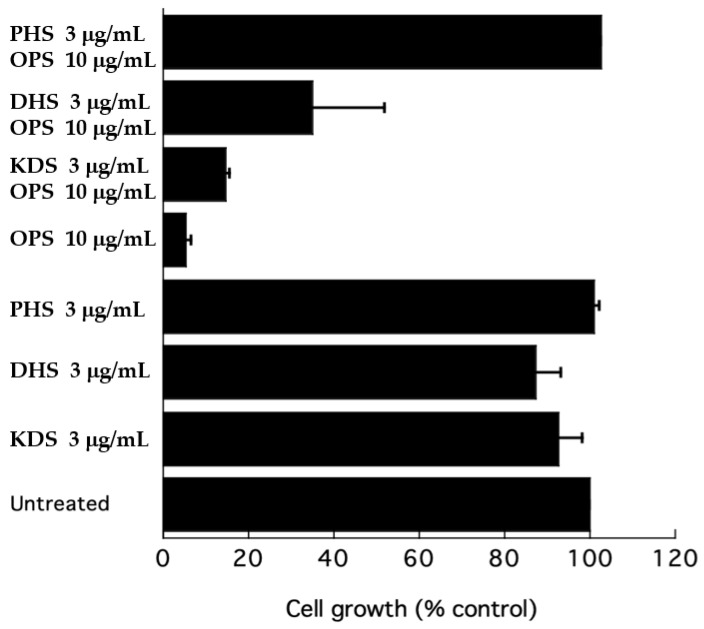
Effect of ketodihydrosphingosine (KDS), dihydrosphingosine (DHS), and phytosphingosine (PHS) on the growth inhibition of OPS against *C. glabrata*. Cells were resuspended in fresh SD dextrose liquid medium and grown for 24 h at 37 °C. Growth after 24 h was measured as absorbance at 600 nm. Error bars indicate the standard error of three experiments.

To further test the effect of OPS on sphingolipid biosynthesis, a growth inhibitory assay was performed in the presence of long-chain bases (LCBs) to test the efficiency of the latter compounds as competitors to rescue cells from inhibition by OPS. A growth inhibitory assay was conducted using ketodihydrosphingosine (KDS), dihydrosphingosine (DHS), and phytosphingosine (PHS) as exogenous sphingoid bases. At 3 μg/mL, KDS, DHS, and PHS were slight inhibitory to *C. glabrata* (Figure 8). However, when added to OPS, these three sphingoid bases showed different effects. The KDS showed no effect on the antifungal activity of OPS. DHS partially reversed the antifungal activity of OPS, showing a 35% cell growth. However, the cells were not viable after 24 h of treatment. In the presence of phytosphingsosine, the antifungal activity of OPS was completely eliminated. Cultures treated with OPS and phytosphingosine showed > 99% cell growth and these cells were viable after 24 h of exposure.

Sphingolipid biosynthesis is essential in yeast and other fungi [24,25,26]. Yeasts are capable of utilizing exogenous long-chain sphingoid bases as substrates for the biosynthesis of sphingolipids when added to culture, which enhances cells survival in times when endogenous sphingoid bases are not available. For example, the cells of *S. cerevisiae lcb* 1 mutants are not viable in the absence of an exogenous supply of sphingoid bases [47]. These long-chain sphingoid bases are converted to *O*-phosphate esters, which are alternative substrates for synthesis of complex phosphoinositides [25,47]. This mechanism led us to ask the question of whether supplementation of exogenous long-chain sphingoid bases could reverse the inhibitory effect of OPS and rescue *C. glabrata* in culture. Our tests showed that PHS, but not KDS and DHS, was able to rescue the cells against the inhibitory activity of OPS. This result indicates that the initial steps in sphingolipid biosynthesis were not affected by OPS, specifically the reactions involved in the assembly of KDS, by condensation of palmitoyl CoA with serine, or the subsequent NADPH-dependent reduction of KDS to DHS. Rather, OPS targets a downstream step in phytoceramide biosynthesis because PHS completely reverses the antifungal activity of OPS.

### 2.4. Oceanapiside (OPS) Induces the Accumulation of Phytosphingosine

**Figure 9:**
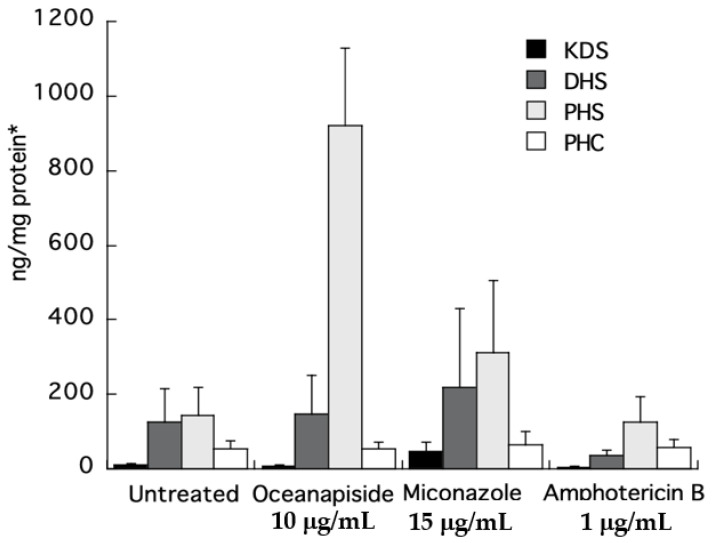
LC-ESI-MS quantification of sphingolipid metabolites obtained from the crude extracts of *C. glabrata* cells treated with oceanapiside, miconazole, and amphotericin B using positive Electrospray Ionization (ESI )and selected ion monitoring (SIM; per ng/cell protein). Error bars indicate the standard error of three experiments. *Absolute protein content of each treatment was found to be in the same range (ca 2 mg/100 μL cell lysate).

To directly identify the steps in sphingolipid biosynthesis affected by OPS, we quantified the long-chain sphingoid bases and ceramides from the crude extracts of the drug-treated cells by comparison with authentic standards using LC-ESI-MS. Quantitative measurements of intermediates in sphingolipid biosynthesis were carried out by selected ion monitoring (SIM) and detection of positive pseudomolecular ions [M + H]^+^ for KDS, DHS, PHS, and PHC, a C_24_-phytoceramide. Two other antifungal agents, miconazole and amphotericin B, with different mechanisms of antifungal activity, were used as control in this assay. The KDS, DHS, and PHC levels were nearly the same for all the treated samples. The most striking result was the increase in phytosphingosine level observed in the cell extracts of OPS-treated cells (Figure 9). The increase was 6.5-fold compared to the untreated cells, or cells treated with miconazole or amphotericin B. The observed cellular PHC level that remained unaltered relative to other treatments in Figure 9 can be explained by de novo synthesis of PHC (phytoceramide) from DHC (dihydroceramide) via sphinganine C4-hydroxylase, SUR2 [29,30,31].

From the foregoing results in Section 2.3 and Section 2.4, we propose two possible targets of OPS: the conversion of DHS to PHS, or PHS to phytoceramide (refer to Figure 1). It is possible that OPS targets the pathway converting DHS to PHS, because even though the cells could no longer synthesize PHS in the presence of OPS, synthesis of complex sphingolipids would still progress, augmented by the supply of exogenous PHS. Alternatively, it is also possible that OPS is a competitive inhibitor of the yeast enzyme that converts PHS to phytoceramide. In the latter scenario, exogenous PHS may reverse the antifungal activity of OPS by displacing it from the enzyme, allowing progression of sphingolipid synthesis. To clarify the details of biosynthetic intervention of OPS, we carried out quantitative analysis of the long-chain sphingoid bases and ceramides in drug-treated *C. glabrata* by LCMS analysis of crude cell-extracts. The concentrations of key long-chain bases in the extracts were measured by LC-ESI-MS. Two other antifungal agents of differing modes of action, miconazole (inhibition of 14α-demethylase in ergosterol biosynthesis) [13] and amphotericin B (ergosterol-dependent cell membrane disruption) [13], were used as controls in this assay. Our results clearly show that PHS accumulates in the OPS-treated cells. The elevated levels of PHS confirms that OPS affects the pathway at a step downstream of PHS synthesis, which is catalyzed by ceramide synthase, i.e., OPS inhibits one or all of LAG1, LAC1, and LIP1 (Figure 1). The increase in phytosphingosine level in OPS-treated cells also differentiates the mechanism of action of OPS from those of miconazole and amphotericin. This highly-sensitive quantitative biochemical assay, which allows direct detection of changes in levels of phytosphingolipids, also validates the hypothesis we proposed in the long-chain sphingoid base rescue experiment: OPS does not target the conversion of DHS to PHS. Instead, the reversal of antifungal activity of OPS by PHS is best explained by competition between the two as substrates for the active site of ceramide synthase which catalyzes the acylation of long chain bases, such as phytosphingosine, to form the corresponding *N*-fatty acyl derivatives, e.g., phytoceramide [26].

The exceptional activity of OPS is likely attributed to the “two-headed” sphingolipid structure and associated multivalency, as reported earlier in our earlier structure-activity study of OPS and related long-chain α,β-diamines and synthetic mono-aminoalkanols [34]. Future work to measure the ceramide synthase activity in the presence and absence of OPS would test the hypothesis that OPS is a competitive inhibitor of ceramide synthase. It is interesting to note that ceramide synthase is the target enzyme of fumonisins, mycotoxins from *Fusarium moniliforme* that are also sphingoid base analogues [48].

These results may have significant implications in the ecological role of oceanapiside and related two-headed sphingolipids in the context of antibiosis against marine-borne fungi and fitness of the host sponges that produce them within the marine environment. The role of antifungal aminoalcohols in these invertebrates has been the subject of speculation [49]. However, basic data, including susceptibilities of the natural products against defined pathogenic marine fungal species, are sparse, and the field remains underexplored.

## 3. Materials and Methods

### 3.1. Animal Materials and Natural Products

Oceanapiside (OPS, > 95% *w*/*w*) was separated from methanol extracts of the sponge, *Oceanapia phillipensis* (Dendy), and purified according to our published procedures [32,33]. Oceanalin (> 95% *w*/*w*) was derived from OPS by acid-promoted methanolysis (HCl, MeOH, and 80 °C), and purified by silica-gel chromatography (12:18:1 methanol/NH_4_OH/chloroform) according to our published procedures [32,33]”.

### 3.2. Media, Yeast Strain, and Culture Conditions

A fluconazole-resistant clinical isolate of *C. glabrata* (University of California Davis Medical Center, UCDMC) was used in the assays. Yeast cells were grown on sabouraud dextrose (SD) agar (glucose 40 g/L, peptone, 10 g/L, and agar 15 g/L) or sabouraud liquid medium (pancreatic digest of casein 5 g/L, peptic digest of fresh meat 5 g/L, and glucose 20 g/L) and incubated at 37 °C.

### 3.3. Time-Dependent Susceptibility Assay

An overnight culture of cells was diluted with fresh SD medium to an OD_600_ of 0.10 to 0.120, and OPS (1 to 30 μg/mL) was added. The cell culture was incubated at 37 °C, and growth rates were determined by measuring the optical density (OD) at 600 nm as a function of time using a Molecular Devices (San Jose, CA, USA) Spectramax Plus plate reader. An aliquot of the OPS-treated cells was taken hourly, re-plated onto fresh SD agar, and incubated for 24 h at 37 °C to check for cell viability.

### 3.4. Live and Dead Susceptibility Assay

An overnight culture of cells was diluted with fresh SD medium to an OD_600_ of 0.10 to 0.120. OPS(10 μg/mL) was added and the cells were incubated for 2 h. The cells were harvested and stained with yeast live/dead kit according to the manufacturer’s instructions (Molecular Probes, Eugene, OR, USA). Briefly, the cells were resuspended in 1 mL of sterile water containing 2% d-(+)-glucose, 10 mM Na-HEPES (pH 7.2), and 5 μM of FUN1 dye. The mixture was incubated at 30 °C in the dark for 30 min and was counterstained with 25 μM Calcofluor White M2R. Five μL of stained cells were immobilized on a clean glass slide and cover slip in preparation for microscopy.

### 3.5. Morphological Studies of Yeast Cells

The morphology of yeast cells treated with OPS (10 μg/mL) was viewed with a photomicroscope (Nikon) using a 100× objective. Triplicate 100 μL aliquots were taken at time points, *t* = 0 to 9 h after incubation. From these samples, at least 500 cells were observed and classified according to the following budding patterns: A, unbudded cells; B, cells with a single small bud; C, cells with a single medium-sized bud; and D, cells with a single large bud. To determine the viability of different budding patterns in OPS-treated cells, a 100 μL of the culture was prepared in triplicates and mixed with an equal volume of methylene blue (Sigma, St. Louis, MO, USA; 0.1 mg/mL in 0.1 M phosphate buffer, pH 7.2). The mixture was incubated for 5 min at room temperature and observed under the microscope.

### 3.6. Actin and Nucleus Staining of C. glabrata

The effect of marine natural products on the organization of actin assembly was determined by using fluorescent probes to visualize the actin cytoskeleton. Briefly, a 100 μL of an overnight culture of *C. glabrata* was transferred to 1 mL of fresh SD medium and incubated at 37 °C to low log (5 × 10^6^/mL). The cell culture was then combined with 10 μg/mL OPS, or 1% DMSO, and incubated for 2 h. After incubation, the cells were fixed in the medium with 4% formaldehyde (methanol free; Ted Pella, Inc., Redding, CA, USA) for 10 min. The cells were harvested (9000 × g for 5 min) and resuspended in 4% formaldehyde for 1 h with frequent mixing, followed by washing (× 2) with phosphate buffered solution, PBS (pH 7.5). The fixed cells were resuspended in 100 µL of PBS and stained with rhodamine-phalloidin, R415 (Molecular Probes, Eugene, OR, USA; 300 units/mL in methanol). The cells were incubated in the dark for 1 h with constant mixing, washed five times with 1 mL PBS, and resuspended in 500 µL of PBS. The mixture was incubated in the dark for 30 min with frequent agitation and washed five times with 1 mL PBS. Rhodamine-phalloidin stained cells (2 μL) were immobilized between a clean glass slide and cover slip for microscopic examination.

### 3.7. Microscopy and Imaging Analysis

Stained cells were viewed with an Olympus IX70 Deltavision Microscope using a 60× 1.4 N.A. objective and illuminated with a 100 W mercury lamp. The following excitation wavelengths were used for actin and nucleus staining: DAPI, 360 and Rhodamine, 555. Yeast cells stained with FUN1 and Calcofluor White M2R used multi-pass filter sets with the following excitation wavelengths: λ 360, 490, and 555 nm. Images were collected in 0.2 µm sections. Two- and three-dimensional light microscopy data collection and computational removal of out-of-focus information were achieved using an integrated, cooled CCD-based, fluorescence light microscopy data collection, processing, and visualization workstation (Applied Precision, Inc. Issaquah, WA, USA) in the Molecular and Cellular Biology Imaging Facility, University of California, Davis. Three-dimensional data sets were processed using Delta Vision’s iterative, constrained three-dimensional deconvolution method.

### 3.8. Long-chain Bases(LCBs) Rescue Experiment

An overnight culture of cells was diluted with fresh SD medium to an OD_600_ of 0.10 to 0.120. The cells were treated with a mixture of OPS (10 μg/mL) and ketodihydrosphingosine (3 μg/mL), OPS (10 μg/mL), and dihydrosphingosine (3 µg/mL), or OPS (10 µg/mL) and phytosphingosine (3 µg/mL), then incubated at 37 °C. Growth rates were measured after 24 h by measuring the optical density at 600 nm using a Molecular Devices Spectramax Plus plate reader. An aliquot of the treated cells was streaked onto fresh SD agar and incubated for 24 h at 37 °C to determine viability and fungicidal versus fungistatic concentrations. Ketodihydrosphingosine, dihydrosphingosine, and phytosphingosine were obtained from Matreya LLC (Pleasant Gap, PA, USA).

### 3.9. Preparation of Cell Extracts

An overnight culture of *C. glabrata* cells was diluted with fresh SD medium to an OD_600_ of 0.40 to 0.410 to make a 1 mL culture. The cells were treated with OPS (10 μg/mL), miconazole (15 μg/mL), and amphotericin B (1 μg/mL) and incubated for 3–4 h at 37 °C. The treated cell extracts were prepared as described previously [50]. Briefly, the cells were harvested, centrifuged (4000 × g for 10 min at 4 °C), and washed three times with sterile water. The cell pellet was resuspended in sterile water with 5% trichloroacetic acid (TCA; final concentration) for 10 min on ice. The cells were then washed once with cold 5% TCA and centrifuged (4000 × g for 10 min at 4 °C) and washed three times with sterile water. The pellet was resuspended in 1 mL methanol/chloroform (1:1) and spiked with 10 μL of 10 μg/mL of 2-amino-heptadecane-1,3-diol, C_17_-DHS (C_17_H_37_NO_2_), prepared by synthesis in our laboratory as a non-natural surrogate internal standard (Supplementary Material, Figure S1). The mixture was sonicated in an ultrasound bath for 10 min and further incubated in a 65 °C bath for 30 min before centrifugation (9000 × g for 10 min). The supernatant was collected, and the pellet was extracted with 1 mL methanol/chloroform (1:1) and twice with 500 μL methanol alone. The pooled extract supernatants were dried and dissolved in 100 μL of acetonitrile and 5% tetrahydrofuran (THF) containing 1 μg/mL (2-amino-4-phenylsulfonyl-1,3-dihydroxyheptadecane and C_23_H_41_NO_4_S) as a secondary synthetic internal standard (IS; Supplementary Material, Figure S1). Stock solutions of authentic standards (ketodihydrosphingosine (KDS), dihydrosphingosine (DHS), phytosphingosine (PHS), and C_24_-phytoceramide (PHC) were prepared by dissolving an appropriate amount of each compound in methanol. Serial dilution of these authentic samples was prepared in acetonitrile containing 5% THF. Protein content of the lysate was determined with the bicinchoninic acid (BCA) assay, as described previously [51]. Quantification of LCBs and ceramides were made by LC-ESI-MS, integration of selected ion-currents and comparison of retention times of the analytes with the authentic standards.

### 3.10. High Performance Liquid Chromatography and Electrospray Ionization Mass Spectrometric (HPLC-ESI-MS) Analysis of LCBs and Sphingolipids

Analysis of sphingolipids and LCBs was carried out using a method we developed for the purpose using LC-ESI-MS of crude cell extracts. A reversed-phase chromatographic separation of the crude cell extracts was carried out on a Luna 3 μm C_18_ 100 Å column (100 × 2.0 mm; Phenomenex, Torrance, CA, USA) on an Agilent 1100 Series liquid chromatography system comprising of a quaternary pump with automatic sampling injector. The gradient components were as follows: H_2_O containing 0.05% formic acid (solvent A), methanol with 0.05% formic acid (solvent B), and 10% THF in methanol with 0.05% formic acid (solvent C). The gradient program was as follows: 0–2 min 50:50 A/B, 2–20 min 3:97 A/B, 20–25 min 50:50 B/C, 25–32 min 50:50 B/C, 32–35 min 90:10 A/B, and 35–38 min 50:50 A/B. The flow rate was set at 0.4 mL/min and the injection volume was 5 μL. The ESI-MS analyses were conducted on a Surveyor MSQ™ MS detector system with an ESI interface (ThermoFinnigan, San Jose, CA, USA). Data acquisition, and qualitative and quantitative analyses were carried out using Xcalibur™ software (ThermoFinnigan, San Jose, CA, USA). For electrospray ionization, the probe temperature was set at 350 °C with cone voltage set at 90 V and a nitrogen flow setting of 4–5 bar. The mass spectrometer was operated in the selected ion monitoring (SIM) mode with positive ion detection (M + H)^+^ of ketodihydrosphingosine, KDS (*m*/*z* 300.28), dihydrosphingosine, DHS (*m*/*z* 302.30), phytosphingosine, PHS (*m*/*z* 317.55), C_24_-phytoceramide, PHC (*m*/*z* 668.7), C_17_-DHS (*m*/*z* 288.2), and C_17_-DHS phenylsulfone (*m*/*z* 428.2) at a total scan duration of 0.4 s. Quantitative evaluations were based on the peak area ratios of each analyte to that of an internal standard, and the calculated amount was normalized based on the known amount of the surrogate standard.

### 3.11. NMR Spectrscopy

NMR samples were prepared in specified deuterated solvents (CDCl_3_ or CD_3_OD, 99.8 atom%, Cambridge Isotope Laboratories, Tewksbury, MA, USA). ^1^H NMR and ^13^C NMR spectra were acquired on a Varian Inova-400 (Palo Alto, CA, USA) operating at frequencies of 399.770 and 100.531 MHz, respectively, using a basic ‘1pulse’ sequence: ~45° π/4 excitation pulse, delay time, d1 = 1.5 s, and broad-band ^1^H decoupling for ^13^C NMR. Data (FIDs) were processed using native software (VNMR, Varian, Palo Alto, CA, USA). ^1^H and ^13^C NMR chemical shifts, δ, are referenced to residual CHCl_3_ (7.26 ppm) and CDCl_3_, (77.16 ppm), respectively.

## 4. Conclusions

In summary, our findings demonstrate that oceanapiside targets the sphingolipid pathway in the pathogenic yeast *Candida glabrata*. Supporting evidence from treatment of *C. glabrata* cell cultures with OPS includes inhibition of polarized growth and budding, disorganization of actin assembly, reversal of antifungal activity by phytosphingosine, and accumulation of phytosphingosine. To date, OPS is the first bifunctional sphingolipid antifungal marine natural product described to inhibit sphingolipid synthesis, specifically at the step converting phytosphingosine to phytoceramide. Future study involving cytotoxicity testing could be undertaken to validate the safety of OPS as an antifungal candidate, but, in all likelihood, synthetic analogues should be pursued for more “drugable” therapeutics.

## Data Availability

The data presented in this study are available in supplementary material.

## Supplementary Materials

The following are available online at https://www.mdpi.com/1660-3397/19/3/126/s1, Figure S1: Synthesis of Internal Standard, experimental details of synthetic procedures, ^1^H and ^13^C NMR spectra of products and representative LCMS data.

## References

[1] Cacaci M., Menchinelli G., Torelli R., Sanglard D., Sanguinetti M., Posteraro B. (2020). New Data on the In Vitro Activity of Fenticonazole against Fluconazole-Resistant *Candida* Species. Antimicrob. Agents Chemother..

[2] Sadeghi G., Ebrahimi-Rad M., Mousavi S., Shams-Ghahfarokhi M., Razzaghi-Abyaneh M. (2018). Emergence of non-*Candida* albicans species: Epidemiology, phylogeny and fluconazole susceptibility profile. J. Med. Mycol..

[3] Hokken M.W., Zwaan B., Melchers W., Verweij P. (2019). Facilitators of adaptation and antifungal resistance mechanisms in clinically relevant fungi. Fungal Genet. Biol..

[4] Nguyen M.H., Peacock J.E., Morris A.J., Tanner D.C., Snydman D.R., Wagener M.M., Rinaldi M.G., Yu V.L. (1996). The changing face of candidemia: Emergence of non-*Candida albicans* species and antifungal resistance. Am. J. Med..

[5] Vazquez J.A., Sobel J.D., Peng G., Steele-Moore L., Schuman P., Holloway W., Neaton J.D. (1999). Evolution of vaginal *Candida* species recovered from human immunodeficiency virus-infected women receiving fluconazole prophylaxis: The emergence of *Candida glabrata*? Terry Beirn Community Programs for Clinical Research in AIDS (CPCRA). Clin. Infect. Dis..

[6] Gale A.N., Sakhawala R.M., Levitan A., Sharan R., Berman J., Timp W., Cunningham K.W. (2020). Identification of Essential Genes and Fluconazole Susceptibility Genes in *Candida glabrata* by Profiling Hermes Transposon Insertions. G3: Genes Genomes Genet..

[7] Pfaller M.A., Diekema D.J. (2007). Epidemiology of Invasive Candidiasis: A Persistent Public Health Problem. Clin. Microbiol. Rev..

[8] Diekema D., Arbefeville S., Boyken L., Kroeger J., Pfaller M. (2012). The changing epidemiology of healthcare-associated candidemia over three decades. Diagn. Microbiol. Infect. Dis..

[9] Fidel P.L., Vazquez J.A., Sobel J.D. (1999). *Candida glabrata*: Review of Epidemiology, Pathogenesis, and Clinical Disease with Comparison toC. albicans. Clin. Microbiol. Rev..

[10] Barchiesi F., Spreghini E., Maracci M., Fothergill A.W., Baldassarri I., Rinaldi M.G., Scalise G. (2004). In Vitro Activities of Voriconazole in Combination with Three Other Antifungal Agents against *Candida glabrata*. Antimicrob. Agents Chemother..

[11] Bolotin-Fukuhara M., Fairhead C. (2016). Editorial: *Candida glabrata*, the other yeast pathogen. FEMS Yeast Res..

[12] Rodrigues C.F., Silva S.C., Henriques M. (2014). *Candida glabrata*: A review of its features and resistance. Eur. J. Clin. Microbiol. Infect. Dis..

[13] Bossche H.V., Willemsens G., Marichal P. (1987). Anti-*Candida* Drugs—The Biochemical Basis for Their Activity. CRC Crit. Rev. Microbiol..

[14] Yang W., Ji X. (2020). Analysis of the microbial species, antimicrobial sensitivity and drug resistance in 2652 patients of nursing hospital. Heliyon.

[15] Shadkchan Y., Segal E. (1999). Antifungal activity of amphotericin B–lipid admixtures in experimental systemic candidosis in naive mice. J. Antimicrob. Chemother..

[16] Nagiec M.M., Young C.L., Zaworski P.G., Kobayashi S.D. (2003). Yeast sphingolipid bypass mutants as indicators of antifungal agents selectively targeting sphingolipid synthesis. Biochem. Biophys. Res. Commun..

[17] Nagiec M.M., Nagiec E.E., Baltisberger J.A., Wells G.B., Lester R.L., Dickson R.C. (1997). Sphingolipid synthesis as a target for antifungal drugs. Complementation of the inositol phosphorylceramide synthase defect in a mutant strain of *Saccharomyces cerevisiae* by the AUR1 gene. J. Biol. Chem..

[18] Healey K.R., Challa K.K., Edlind T.D., Katiyar S.K. (2015). Sphingolipids Mediate Differential Echinocandin Susceptibility in *Candida albicans* and *Aspergillus nidulans*. Antimicrob. Agents Chemother..

[19] Kumar M., Singh A., Kumari S., Kumar P., Wasi M., Mondal A.K., Rudramurthy S.M., Chakrabarti A., Gaur N.A., Gow N.A. (2021). Sphingolipidomics of drug resistant *Candida auris* clinical isolates reveal distinct sphingolipid species signatures. Biochim. et Biophys. Acta (BBA) Mol. Cell Biol. Lipids.

[20] Healey K.R., Katiyar S.K., Raj S., Edlind T.D. (2012). CRS-MIS in *Candida glabrata*: Sphingolipids modulate echinocandin-Fks interaction. Mol. Microbiol..

[21] McEvoy K., Normile T.G., Del Poeta M. (2020). Antifungal Drug Development: Targeting the Fungal Sphingolipid Pathway. J. Fungi.

[22] Fernandes C.M., Del Poeta M. (2020). Fungal sphingolipids: Role in the regulation of virulence and potential as targets for future antifungal therapies. Expert Rev. Anti-Infective Ther..

[23] Mor V., Rella A., Farnoud A.M., Singh A., Munshi M., Bryan A.M., Naseem S., Konopka J.B., Ojima I., E Bullesbach E. (2015). Identification of a New Class of Antifungals Targeting the Synthesis of Fungal Sphingolipids. mBio.

[24] Dickson R.C. (1998). Sphingolipid functions in *Saccharomyces cerevisiae*: Comparison to mammals. Annu. Rev. Biochem..

[25] Dickson R.C., Lester R.L. (1999). Metabolism and selected functions of sphingolipids in the yeast *Saccharomyces cerevisiae*. Biochim. et Biophys. Acta (BBA) Mol. Cell Biol. Lipids.

[26] Obeid L.M., Okamoto Y., Mao C. (2002). Yeast sphingolipids: Metabolism and biology. Biochim. et Biophys. Acta (BBA) Mol. Cell Biol. Lipids.

[27] Guillas I., Kirchman P.A., Chuard R., Pfefferli M., Jiang J.C., Jazwinski S.M., Conzelmann A. (2001). C26-CoA-dependent ceramide synthesis of *Saccharomyces cerevisiae* is operated by Lag1p and Lac1p. EMBO J..

[28] Schorling S., Vallée B., Barz W.P., Riezman H., Oesterhelt D. (2001). Lag1p and Lac1p are essential for the Acyl-CoA-dependent ceramide synthase reaction in *Saccharomyces cerevisiae*. Mol. Biol. Cell..

[29] Haak D., Gable K., Beeler T., Dunn T. (1997). Hydroxylation of *Saccharomyces cerevisiae* Ceramides Requires Sur2p and Scs7p. J. Biol. Chem..

[30] Montefusco D.J., Matmati N., Hannun Y.A. (2014). The yeast sphingolipid signaling landscape. Chem. Phys. Lipids.

[31] Jadhav S., Greenberg M.L. (2014). Harnessing the power of yeast to elucidate the role of sphingolipids in metabolic and signaling processes pertinent to psychiatric disorders. Clin. Lipidol..

[32] Nicholas G.M., Hong T.W., Molinski T.F., Lerch M.L., Cancilla M.T., Lebrilla C.B. (1999). Oceanapiside, an antifungal bis- α,ω--amino alcohol glycoside from the marine sponge *Oceanapia phillipensis*. J. Nat. Prod..

[33] Nicholas G.M., Molinski T.F. (2010). Enantiodivergent Biosynthesis of the Dimeric Sphingolipid Oceanapiside from the Marine Sponge *Oceanapia phillipensis*. Determination of Remote Stereochemistry. J. Am. Chem. Soc..

[34] Nicholas G.M., Li R., Macmillan J.B., Molinski T.F. (2002). Antifungal activity of bifunctional sphingolipids. intramolecular synergism within long-chain α,ω-bis-aminoalcohols. Bioorganic Med. Chem. Lett..

[35] Millard P.J., Roth B.L., Thi H.P., Yue S.T., Haugland R.P. (1997). Development of the FUN-1 family of fluorescent probes for vacuole labeling and viability testing of yeasts. Appl. Environ. Microbiol..

[36] Kucsera J., Yarita K., Takeo K. (2000). Simple detection method for distinguishing dead and living yeast colonies. J. Microbiol. Methods.

[37] Friant S., Lombardi R., Schmelzle T., Hall M.N., Riezman H. (2001). Sphingoid base signaling via Pkh kinases is required for endocytosis in yeast. EMBO J..

[38] Cheng J., Park T.-S., Fischl A.S., Ye X.S. (2001). Cell Cycle Progression and Cell Polarity Require Sphingolipid Biosynthesis in *Aspergillus nidulans*. Mol. Cell. Biol..

[39] Endo M., Takesako K., Kato I., Yamaguchi H. (1997). Fungicidal action of aureobasidin A, a cyclic depsipeptide antifungal antibiotic, against *Saccharomyces cerevisiae*. Antimicrob. Agents. Chemother..

[40] Dickson R.C., Lester R.L. (2002). Sphingolipid functions in *Saccharomyces cerevisiae*. Biochim. et Biophys. Acta (BBA) Mol. Cell Biol. Lipids.

[41] Dickson R.C., Sumanasekera C., Lester R.L. (2006). Functions and metabolism of sphingolipids in *Saccharomyces cerevisiae*. Prog. Lipid Res..

[42] Dickson R.C. (2010). Roles for Sphingolipids in *Saccharomyces cerevisiae*. Retin. Degener. Dis..

[43] Karpova T.S., McNally J.G., Moltz S.L., Cooper J.A. (1998). Assembly and Function of the Actin Cytoskeleton of Yeast: Relationships between Cables and Patches. J. Cell Biol..

[44] Gihana G.M., Cross-Najafi A.A., Lacefield S. (2021). The mitotic exit network regulates the spatiotemporal activity of Cdc42 to maintain cell size. J. Cell Biol..

[45] Garabedian M.V., Wirshing A., Vakhrusheva A., Turegun B., Sokolova O.S., Goode B.L. (2020). A septin-Hof1 scaffold at the yeast bud neck binds and organizes actin cables. Mol. Biol. Cell.

[46] Akram Z., Ahmed I., Mack H., Kaur R., Silva R.C., Castilho B.A., Friant S., Sattlegger E., Munn A.L. (2020). Yeast as a Model to Understand Actin-Mediated Cellular Functions in Mammals—Illustrated with Four Actin Cytoskeleton Proteins. Cells.

[47] Buede R., Rinker-Schaffer C., Pinto W.J., Lester R.L., Dickson R.C. (1991). Cloning and characterization of LCB1, a *Saccharomyces* gene required for biosynthesis of the long-chain base component of sphingolipids. J. Bacteriol..

[48] Wang E., Norred W.P., Bacon C.W., Riley R.T., Merrill A.H. (1991). Inhibition of sphingolipid biosynthesis by fumonisins. Implications for diseases associated with Fusarium moniliforme. J. Biol. Chem..

[49] Molinski T. (2004). Antifungal Compounds from Marine Organisms. Curr. Med. Chem. Anti-Infective Agents.

[50] Lester R.L., Dickson R.C. (2001). High-Performance Liquid Chromatography Analysis of Molecular Species of Sphingolipid-Related Long Chain Bases and Long Chain Base Phosphates in *Saccharomyces cerevisiae* after Derivatization with 6-Aminoquinolyl-N-hydroxysuccinimidyl Carbamate. Anal. Biochem..

[51] Smith P., Krohn R., Hermanson G., Mallia A., Gartner F., Provenzano M., Fujimoto E., Goeke N., Olson B., Klenk D. (1985). Measurement of protein using bicinchoninic acid. Anal. Biochem..

