# Supplementary material for: Oceanapiside, a Marine Natural Product, Targets the Sphingolipid Pathway of Fluconazole-Resistant Candida glabrata
Source: Mar Drugs. 2021 Feb 26;19(3):126. doi: 10.3390/md19030126 (PMC7996939; doi:10.3390/md19030126)
Supplement: Supplementary file 1 [file marinedrugs-19-00126-s001.pdf]

SUPPORTING INFORMATION

OCEANAPISIDE, A MARINE NATURAL PRODUCT, TARGETS THE SPHINGOLIPID PATHWAY OF FLUCONAZOLE-RESISTANT *CANDIDA GLABRATA*

Doralyn S. Dalisay,[1,2] Evan W. Rogers[1] and Tadeusz F. Molinski[1,3,*]

1. Department of Chemistry and Biochemistry, University of California, San Diego, 9500 Gilman Drive MC0358, La Jolla, CA 92093, USA
2. Center for Chemical Biology and Biotechnology (C2B2) and Department of Biology, College of Liberal Arts, Sciences and Education, University of San Agustin, Iloilo City, 5000, Philippines
3. Skaggs School of Pharmacy and Pharmaceutical Sciences, University of California, San Diego, 9500 Gilman Drive, La Jolla, CA 92093, USA

Page *Title* *Content*

Page	Title	Content
S1	Scheme S1	Synthesis of internal standard (IS)
S2	Scheme S2	Synthesis of surrogate standard *nor*-dihydrosphingosine (C_{17}-DHS) & Synthetic Experimental Protocols
S6	Figure S1	^{1}H NMR ($CDCl_3$, 400 MHz) of compound **4a**
S7	Figure S2	^{13}C NMR ($CDCl_3$, 100 MHz) of compound **4a**
S8	Figure S3	^{1}H NMR (CD_3OD, 400 MHz) of compound (2*S*,3*R*)-**5**
S9	Figure S4	^{13}C NMR (CD_3OD, 100 MHz) of compound (2*S*,3*R*)-**5**
S10	Figure S5	^{1}H NMR ($CDCl_3$, 400 MHz) of **8** (dr 4:1) major isomer, (2*S*,3*R*)-**8**
S11	Figure S6	^{13}C NMR ($CDCl_3$, 100 MHz) of compound (2*S*,3*R*)-**8**
S12	Figure S7	^{1}H NMR ($CDCl_3$, 400 MHz) of compound (2*S*,3*R*)-**9b**
S13	Figure S8	^{13}C NMR ($CDCl_3$, 100 MHz) of compound (2*S*,3*R*)-**9b**
S14	Figure S9	^{1}H NMR (CD_3OD, 400 MHz) of compound (2*S*,3*R*)-**10** [C_{17}-DHS]
S15	Figure S10	^{13}C NMR (CD_3OD, 100 MHz) of compound (2*S*,3*R*)-**10** [C_{17}-DHS]
S16	Figure S11	LCMS Data – Sphingolipid Standards, DHS & PHS
S17	Figure S12	LCMS Data – Standard Curves, DHS & PHS

Scheme S1. Synthesis of internal standard (IS).*

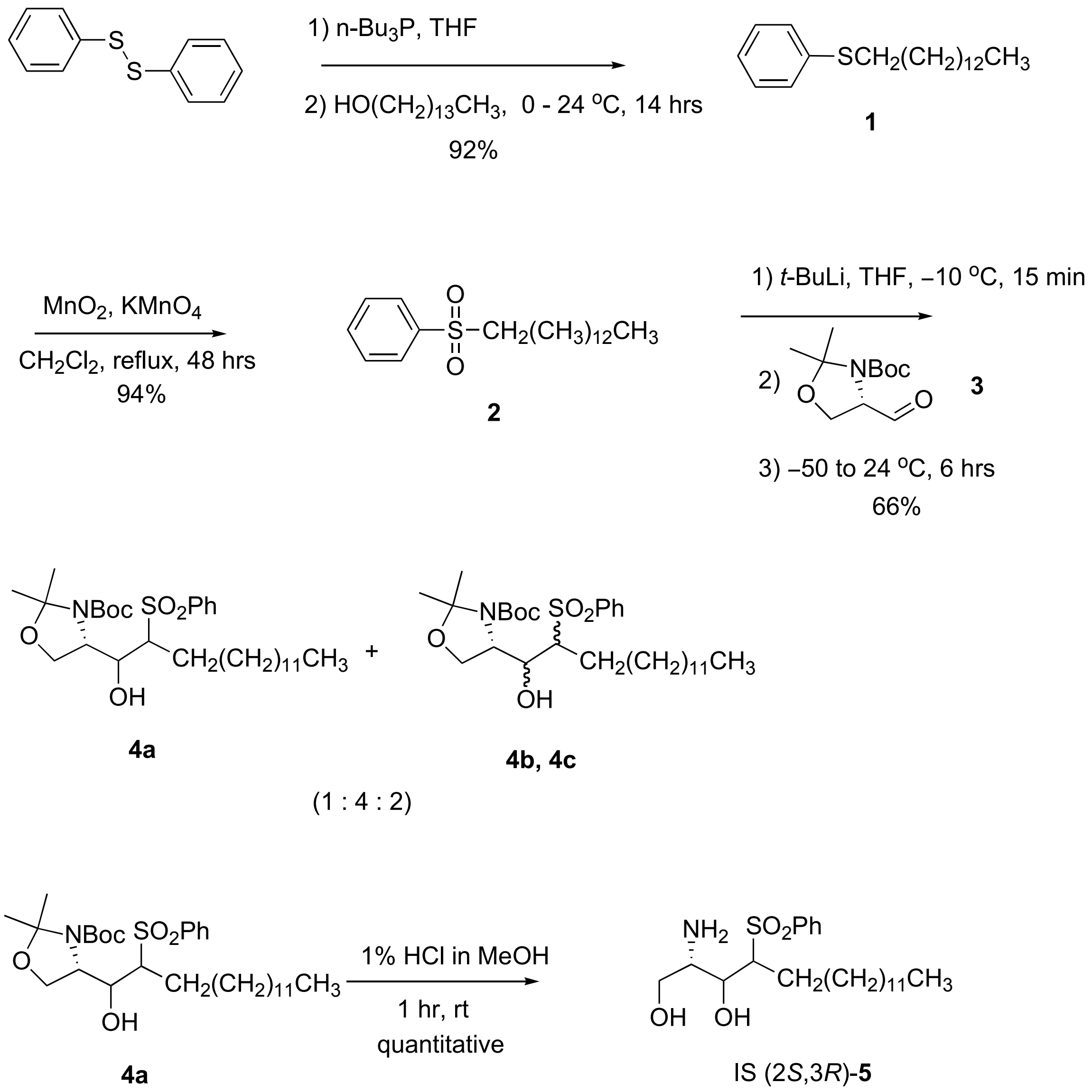

Method references.[1, 2, 3]

*Stereochemical assignment based on known Felkin-Ahn diastereoselectivity of non-chelate controlled addition of nucleophiles to serinal derivatives (Reetz et al).[7] C-4 is unassigned.

Scheme S2. Synthesis of surrogate standard *nor*-dihydrosphingosine (C_{17}-DHS).*

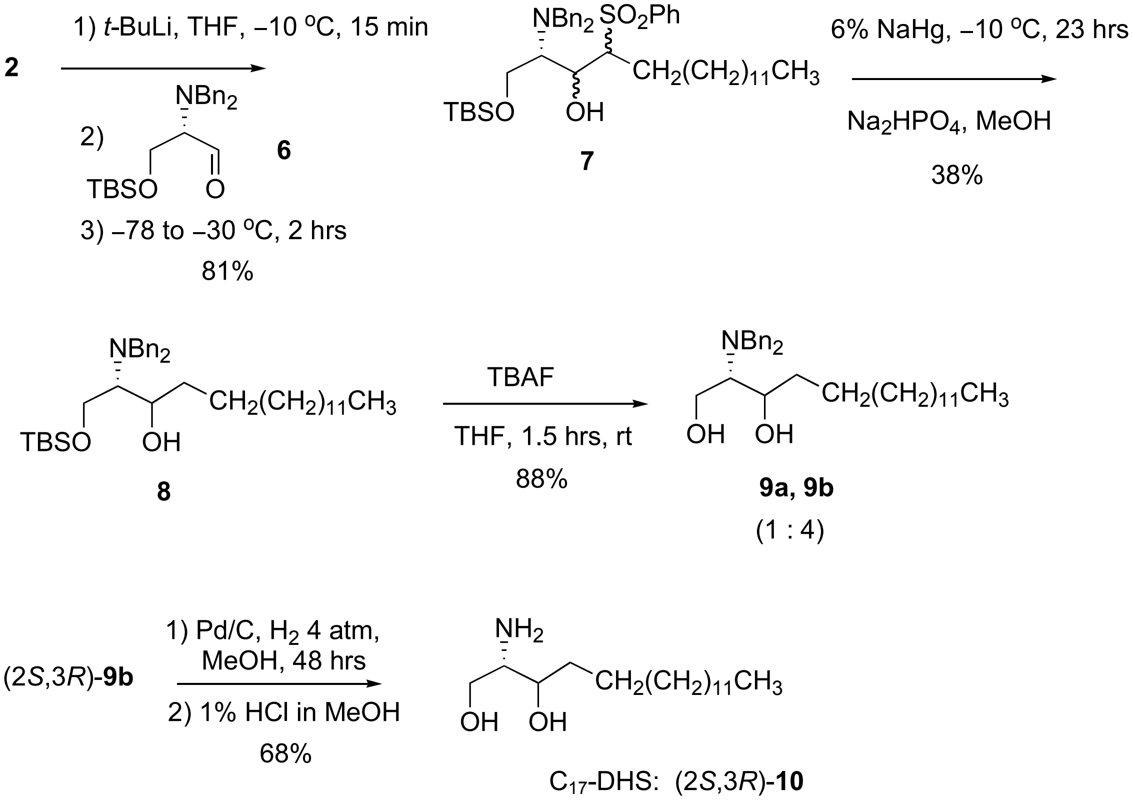

Method references.[4, 5, 6]

*Stereochemical assignment based on known Felkin-Ahn diastereoselectivity of non-chelate controlled addition of nucleophiles to serinal derivatives (Reetz et al).[7]

Phenyl(tetradecyl)sulfane (1). Under an atmosphere of nitrogen, *n*-Bu_3P (7.26 mL, 29.1 mmol) was added dropwise to a solution of diphenyldisulfide (6.36 g, 29.1 mmol) in anhydrous THF at 0 °C. The mixture was stirred for 15 min then tetradecan-1-ol (5.0 g, 23.3 mmol in THF) was added dropwise. The solution was warmed to 24 °C over 24 hrs and quenched with 150 mL water and the mixture was extracted with ethyl ether (5x50 mL) and combined extracts washed with brine (50 mL), dried over $NaSO_4$ and concentrated in vacuo. Flash chromatography (silica, 3% ethyl acetate in hexane) provided **1** (6.35 g, 92%) as white solid. Compound **1** matched literature values.[8]

Phenyl(tetradecyl)sulfone (2). Under an atmosphere of nitrogen, finaly ground $KMnO_4$ (2.5 g, 15.8 mmol) and MnO4 (508 mg, 5.8 mmol) was added to a solution of **1** (1.0 g, 3.26 mmol) in anhydrous dichloromethane. The mixture was refluxed for 2 days then filtered through celite and rotovaped to dryness. Flash chromatography (silica, dichloromethane) provided **2** (1.04 g, 94%) as white solid. Compound **2** matched literature values.[7]

(*S*)-*tert*-butyl 4-1-hydroxy-2-(phenylsulfonyl)pentadecyl)-2,2-dimethyloxazolidine-3-carboxylate (4a). Under an atmosphere of nitrogen, *t*-BuLi
(192 μmol, 1.7 M in pentane) was added dropwise to a solution of **2** (65 mg, 192 μmol) in

anhydrous THF at –10 °C. The mixture was stirred for 15 min then cooled to –50 °C and **3** (43.7 mg, 191 μmol in THF) was added dropwise over 5 min. The solution was warmed to 24 °C over 6 hrs and quenched with 5 mL saturated aqueous NH_4Cl. The mixture was extracted with ethyl ether (5x5 mL) and combined extracts washed with brine (5 mL), dried over $NaSO_4$ and concentrated in vacuo. Flash chromatography (silica, 10% ethyl acetate in hexane then 25% ethyl acetate in hexane) provided **4a**, **4b**, and **4c** (71.5 mg, 66%, 1: 4:2 ratio) as pale yellow viscous oils.:IR (neat) ν 3442, 2925, 2854, 1711, 1498, 1447, 1392, 1366, 1301, 1287, 1246, 1167, 1142, 1081, 847, 727, 690 cm^{-1}; $[\alpha]_D^{22}$ –4.6° ($CHCl_3$, *c* 0.88); ^1H NMR (400 MHz, $CDCl_3$) *δ* .88 (t, *J* = 6.8 Hz, 3H), 1.10-1.30 (bm, 22H), 1.45-1.60 (bm, 16H), 1.79 (bs, 1H), 3.12 (dt, *J* = 10.8, 2.4 Hz, 1H), 3.98 (bm, 1H), 4.14 (bm, 1H), 4.98 (bm, 1H), 5.11 (bs, 1H), 7.54(t, *J* = 7.2 Hz, 2H), 7.62 (d, *J* = 7.2 Hz, 2H), 7.94 (d, *J* = 7.2 Hz, 2H); selected ^{13}C NMR (100 MHz, $CDCl_3$) *δ* 14.3 (CH_3), 22.8 (CH_2), 26.4 (CH_2), 28.4 (CH_3), 28.5 (CH_3), 29.3 (CH_2), 29.5 (CH_2), 29.6 (CH_2), 29.7 (CH_2), 29.8 (CH_2), 32.0 (CH_2), 52.1 (CH), 64.6 (CH_2), 67.5 (CH), 71.0 (CH), 80.1 (C), 99.4 (C), 128.1 (CH), 128.6 (CH), 129.0 (CH), 129.5 (CH), 133.6 (CH), 134.3 (CH), 137.8 (C), 156.0 (C); HRFABMS m/z 568.3662 $[M+H]^+$, calcd. for $C_{31}H_{54}N_1O_6S_1$ 568.3672.

(2*S*,3*R*)-2-amino-4-(phenylsulfonyl)heptadecane-1, 3-diol (5). A solution of **4a** (7.0 mg, 12.3 μmol) in MeOH (1 mL) with 1% HCl was stirred for 1 hour at room temperature. The solution was concentrated under reduced pressure to give the hydrochloride salt of **5** (5.7 mg, quantitative) as a white solid.: IR (neat) ν 3216, 2954, 2923, 2853, 1712, 1586, 1493, 1467, 1446, 1299, 1144, 1083, 759, 730, 689, 655 cm^{-1}; $[\alpha]_D^{22}$ 0.1° (CH_3OH, *c* 0.73); ^1H NMR (400 MHz, CD_3OD) *δ* 0.90 (t, *J* = 6.8 Hz, 3H), 1.15-1.35 (bm, 22H), 1.78 (bm, 2H), 3.41 (m, 1H), 3.86 (dd, *J* = 12.0, 5.2 Hz, 1H), 3.91 (dd, *J* = 12.0, 4.0 Hz, 1H), 4.02 (p, *J* = 4.0 Hz, 1H), 4.18 (dd, *J* = 8.0, 2.4 Hz, 1H), 7.63 (t, *J* = 7.2 Hz, 2H), 7.73 (t, *J* = 7.2 Hz, 1H), 7.95 (d, *J* = 7.2 Hz, 2H); ^{13}C NMR (100 MHz, CD_3OD) *δ* 14.4 (CH_3), 23.7 (CH_2), 27.5 (CH_2), 28.0 (CH_2), 30.0 (CH_2), 30.2 (CH_2), 30.5 (CH_2), 30.6 (CH_2), 30.7 (CH_2), 30.76 (CH_2), 30.8 (CH_2), 33.1 (CH_2), 56.7 (CH), 60.3 (CH_2), 67.3 (CH), 68.4 (CH), 130.1 (CH), 130.3 (CH), 135.0 (CH), 140.9 (C); HRFABMS m/z 428.2831 $[M+H]^+$, calcd. for $C_{23}H_{42}N_1O_4S_1$ 428.2835.

(2*R*)-1-*tert*-butyldimethylsilanyloxy-2-(dibenzylamino)-4-(phenylsulfonyl)heptadecan-3-ol (7). Under an atmosphere of nitrogen, *t*-BuLi (2.04 mL, 3.48 mmol, 1.7 M in pentane) was added dropwise to a solution of **2** (1.10 g, 3.25 mmol) in anhydrous THF at –20 °C. The mixture was stirred for 2 hours then cooled to –78 °C and **6** (1.00 g, 2.60 mmol in THF) was added dropwise over 15 min. The solution was warmed to –30 °C over 2 hrs and quenched with 50 mL saturated aqueous NH_4Cl. The mixture was extracted with ethyl ether (5x50 mL) and combined extracts washed with brine (250 mL), dried over $NaSO_4$ and concentrated in vacuo. Flash chromatography (silica, 10% ethyl acetate in hexane then 25% ethyl acetate in hexane) provided **7** (1.52 g, 81% by NMR, mixture of diastereomers) and starting sulfone **2** as an inseparable viscous oil. Product was not characterized and was used as is in the next step.

(2*S*,3*R*)-1-*tert*-butyldimethylsilanyloxy-2-(dibenzylamino)heptadecan-3-ol (8). Under an atmosphere of nitrogen, 6% NaHg (838 mg, 2.1 mmol) was added to a solution of **7** (330 mg, 0.45 mmol) and Na_2HPO_4 (308 mg, 2.16 mmol) in anhydrous MeOH at –20 °C. The mixture was stirred for 23 hours then the reaction was quenched with 25 mL saturated aqueous NH_4Cl. The mixture was extracted with ethyl ether (5x10 mL) and combined extracts washed with brine (50 mL), dried over $NaSO_4$ and concentrated in vacuo. Flash chromatography (silica, 20% dichloromethane in hexane) provided **8** (76.6 mg, 38%, mixture of diastereomers 1 : 4.8 by NMR) as a viscous oil.: IR (neat) ν 3476, 3085, 3063, 3027, 2953, 2925, 2854, 2803, 1494, 1462, 1360, 1256, 1073, 836, 776, 746, 698 cm^{-1}; $[\alpha]_D^{24}$ 0.6° ($CHCl_3$, *c* 4.56); For major diasteromer ^1H NMR (400 MHz, $CDCl_3$) δ 0.10 (s, 3H), 0.12 (s, 3H), 0.89 (t, *J* = 7.6 Hz, 3H), 0.91 (s, 9H), 1.26 (bm, 25H), 1.67 (m, 1H), 2.66 (q, *J* = 5.2 Hz, 1H), 3.00 (d, *J* = 4.8 Hz, 1H), 3.62 (d, *J* = 13.6 Hz, 2H), 3.83-3.92 (m, 3H), 3.97-4.05 (m, 2H), 7.20-7.35 (m, 10H); ^{13}C NMR (100 MHz, $CDCl_3$) δ -5.48 (CH_3), -5.41 (CH_3), 14.3 (CH_3), 18.2 (C), 22.8 (CH_2), 25.6 (CH_2), 26.0 (CH_3), 29.5 (CH_2), 29.8 (CH_2), 29.9 (CH_2), 32.0 (CH_2), 35.1 (CH_2), 55.4 (CH), 61.3 (CH_2), 61.5 (CH), 72.4 (CH_2), 127.1 (CH), 128.4 (CH), 129.0 (CH), 140.2 (C); HRFABMS m/z 582.4735 $[M+H]^+$, calcd. for $C_{37}H_{64}N_1O_2Si_1$ 582.4706.

(2*S*, 3*R*)-2-(dibenzylamino)heptadecan-1, 3-diol (9b). Under an atmosphere of nitrogen, tetrabutylammonium floride (300 μL, 300 μmol, 1.0 M in THF) was added to a solution of **8** (40 mg, 68.7 μmol) in anhydrous THF at room temperature. The mixture was stirred for 30 min then quenched with 20 mL saturated aqueous NH_4Cl. The mixture was extracted with ethyl ether (5x5 mL) and combined extracts washed with brine (25 mL), dried over $NaSO_4$ and concentrated in vacuo. Flash chromatography (12g Analogix silica column, 20% ethyl acetate in hexane) provided **9a** and **9b** (1 : 4.5) (28.2 mg, 88%) as viscous oils.: Characterization for **9b** IR (neat) ν 3381, 3085, 3062, 3027, 2923, 2853, 2804, 1602, 1494, 1454, 1364, 1250, 1117, 1071, 1027, 747, 698 cm^{-1}; $[\alpha]_D^{23}$ –1.0°
($CHCl_3$, *c* 3.45); ^1H NMR (400 MHz, $CDCl_3$) δ 0.89 (t, *J* = 7.2 Hz, 3H), 1.28 (bm, 24H), 1.65 (bm, 1H), 1.83 (bs, 1H), 2.69 (q, *J* = 5.6 Hz, 1H), 2.81 (bs, 1H), 3.69 (d, *J* = 13.6 Hz, 1H), 3.75-3.85 (bm, 3H), 3.94 (dd, *J* = 11.2, 6.8 Hz, 1H), 4.01 (bs, 1H), 7.21-7.35 (bm, 10H); ^{13}C NMR (100 MHz, $CDCl_3$) δ 14.3 (CH_3), 22.8 (CH_2), 25.6 (CH_2), 29.5 (CH_2), 29.6 (CH_2), 29.7 (CH_2), 29.8 (CH_2), 32.1 (CH_2), 35.9 (CH_2), 54.7 (CH_2), 58.9 (CH_2), 62.3 (CH), 71.3 (CH), 127.3 (CH), 128.5 (CH), 129.1 (CH), 139.7 (C); HRFABMS m/z 468.3844 $[M+H]^+$, calcd. for $C_{31}H_{50}N_1O_2$ 468.3842.

(2*S*, 3*R*)-2-aminoheptadecan-1, 3-diol (10). To **9b** (20 mg, 42.8 μmol) in methanol (1.5 mL) was added Pd on carbon (30 mg, 28 μmol, 10% Pd on activated carbon). The mixture was placed on a Parr hydrogenator under 4 atm of H_2 and shaken for 48 hrs. The solution was filtered through a celite plug and concentrated in vacuo. The residue was redisolved in 1% HCl in methanol and run through a C18 SPE cartridge (1 g) and eluted with 10 mL of 0.5% HCl in acetonitrile : methanol : water (2 : 1 : 1) to obtain the hydrochloride salt of **10** (9.4 mg, 68%) as a viscous oil.: IR (neat) ν 3331, 2917, 2850, 1596, 1497, 1467, 1159, 1124, 1048, 1018, 721 cm^{-1}; $[\alpha]_D^{23}$ 3.9° (CH_3OH, *c* 0.29); ^1H NMR (400 MHz, CD_3OD) δ 0.90 (t, *J* = 7.2 Hz, 3H), 1.29 (bm, 24H), 1.49 (m, 2H), 3.91 (m, 1H), 3.69 (dd, *J* = 11.4, 8.8 Hz, 1H), 3.77 (m, 1H), 3.83 (dd, *J* = 11.4, 3.6 Hz, 1H);

^{13}C NMR (100 MHz, CD_3OD) δ 14.4 (CH_3), 23.7 (CH_2), 27.0 (CH_2), 30.5 (CH_2), 30.6 (CH2), 30.7 (CH_2), 30.73 (CH_2), 30.8 (CH_2), 33.1 (CH_2), 34.2 (CH_2), 58.5 (CH_2), 58.8 (CH), 70.3 (CH); HRFABMS m/z 288.2898 $[M+H]^+$, calcd. for $C_{17}H_{38}N_1O_2$ 288.2903.

---

[1] Shaabani, A.; Mirzaei, P.; Lee, D. G. *Catal. Lett.* **2004**, *97*, 119-123. Dondoni, A.; Perrone, D.

[2] *Org. Synth.* **2000**, *77*, 64-77.

[3] Hart, D. J.; Wu, W.-L. *Tetrahedron Lett.* **1996**, *37*, 5283-5286.

[4] Laieb, T.; Chastanet, J.; Zhu, J. *J. Org. Chem.* 1998, *63*, 1709-1713.

[5] Zhao, X.-Y.; Janda, K. D. *Bioorg. Med. Chem. Lett.* **1998**, *8*, 2439-2442. Shimizu, M.;

[6] Wakioka, I.; Fujisawa, T. *Tetrahedron Lett.* **1997**, *38*, 6027-6030.

[7] Reetz, M. T.; Drews, M. W.; Schmitz, A. *Angew. Chem. Intl. Ed*. **1987**, *26*, 1141-1143.

[8] Findeis, M. A.; Whitesides, G. M. *J. Org. Chem.* **1987**, *52*, 2838-2848.

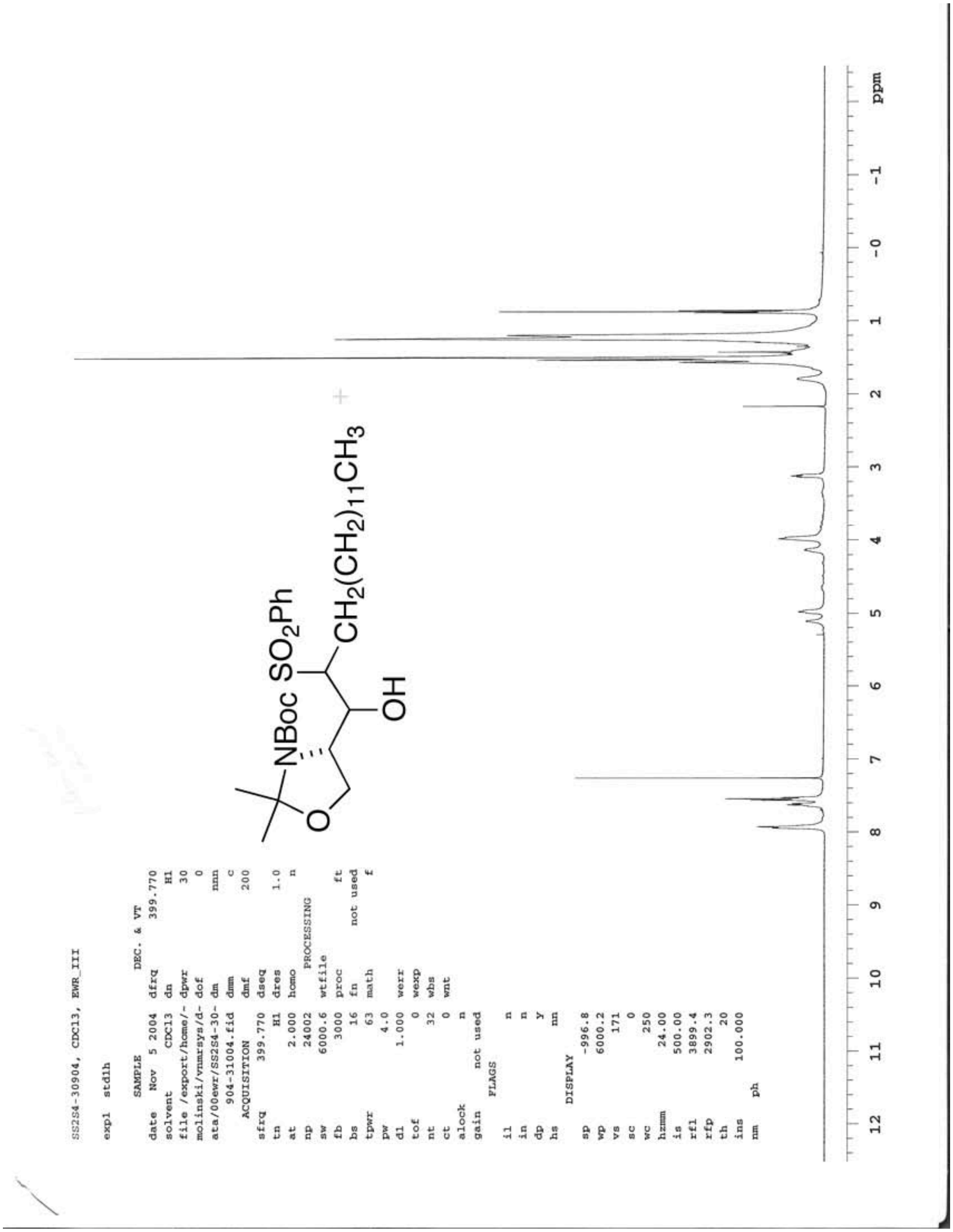

Figure S1: ^{1}H NMR ($CDCl_3$, 400 MHz) of compound **4a**

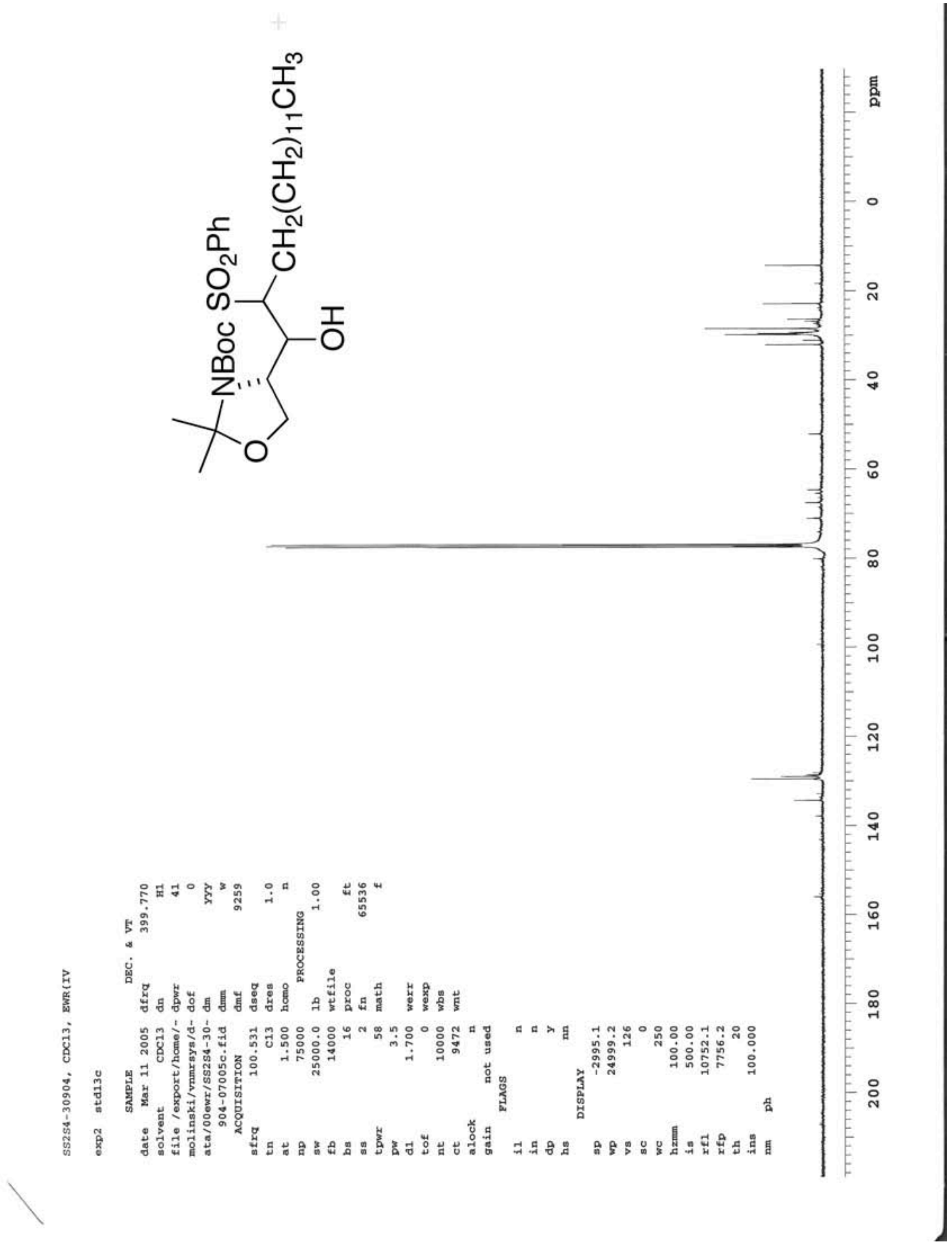

Figure S2: ^{13}C NMR ($CDCl_3$, 100 MHz) of compound **4a**

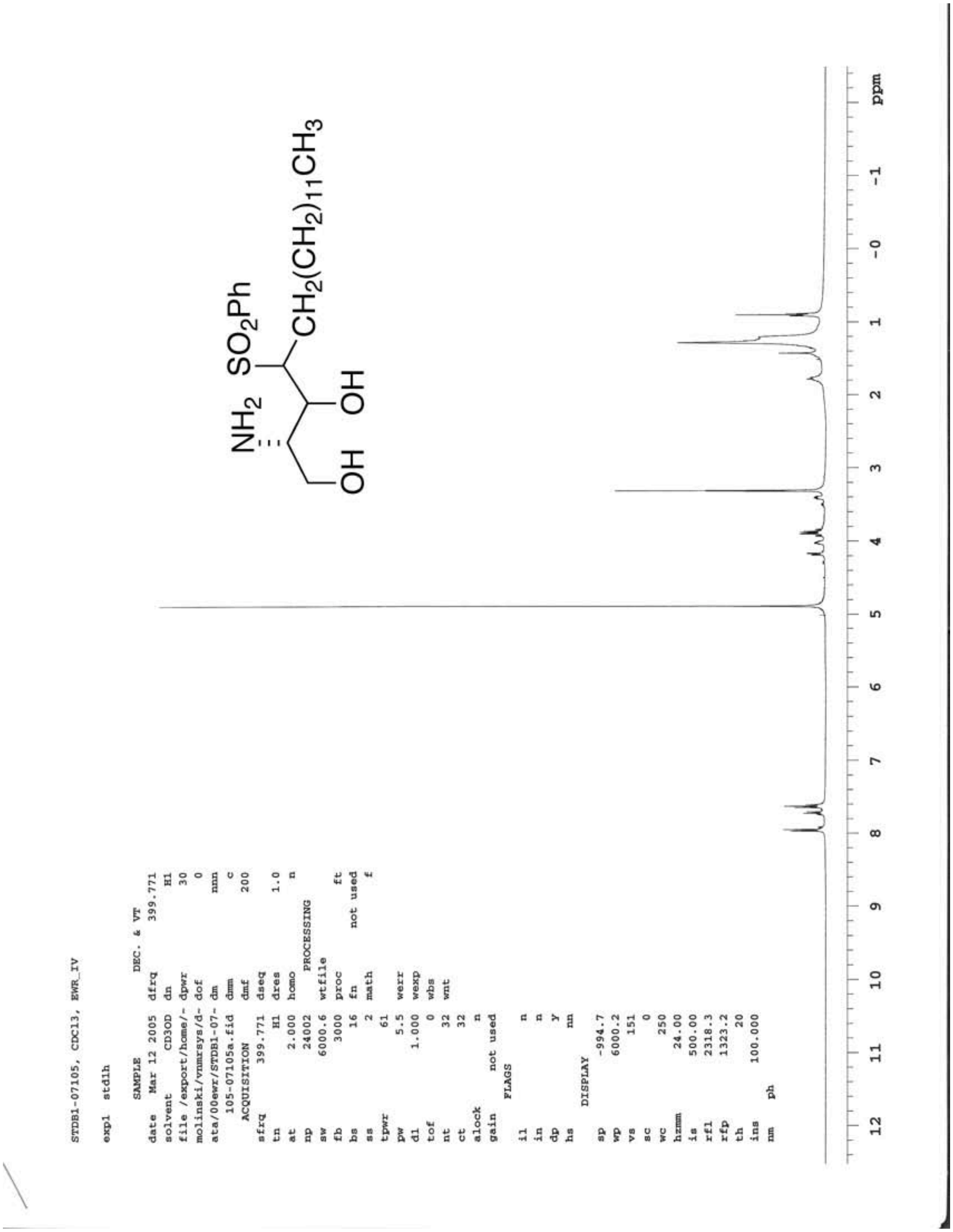

Figure S3: ^{1}H NMR (CD_3OD, 400 MHz) of compound (2*S*,3*R*)-**5**

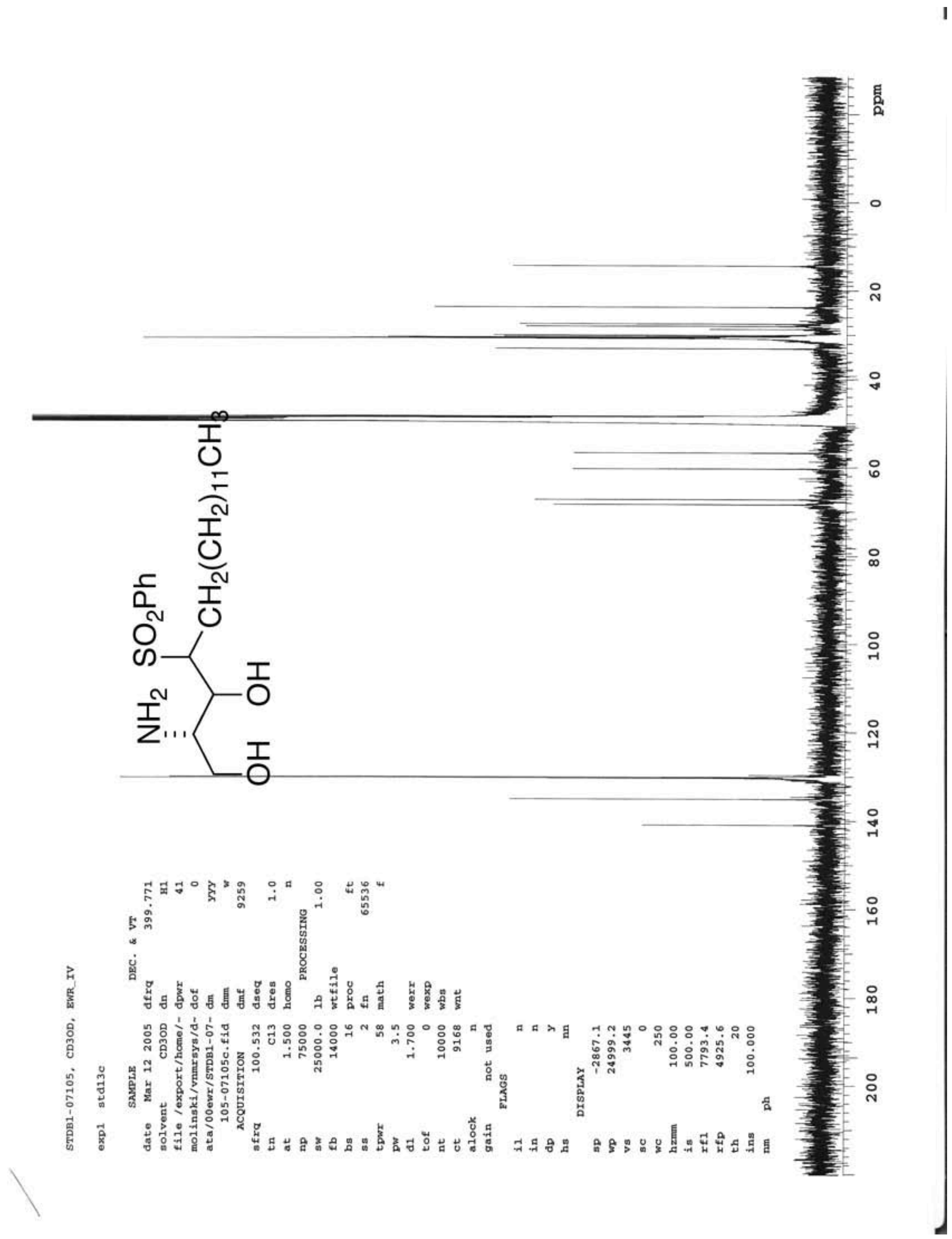

Figure S4: ^{13}C NMR (CD_3OD, 100 MHz) of compound (2*S*,3*R*)-**5**

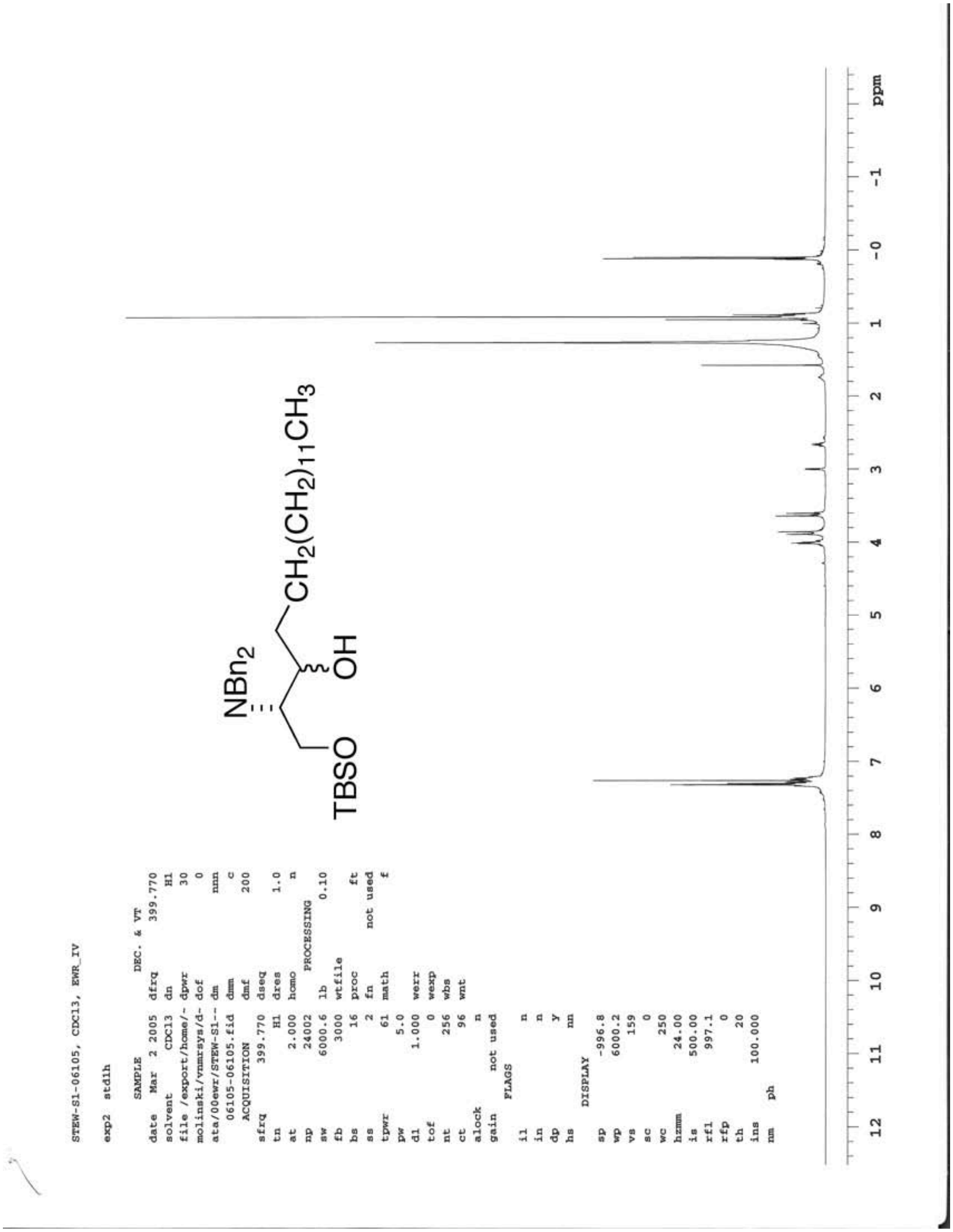

Figure S5: ^{1}H NMR ($CDCl_3$, 400 MHz) of compound (dr 4:1, major isomer, (2*S*,3*R*)**-8**

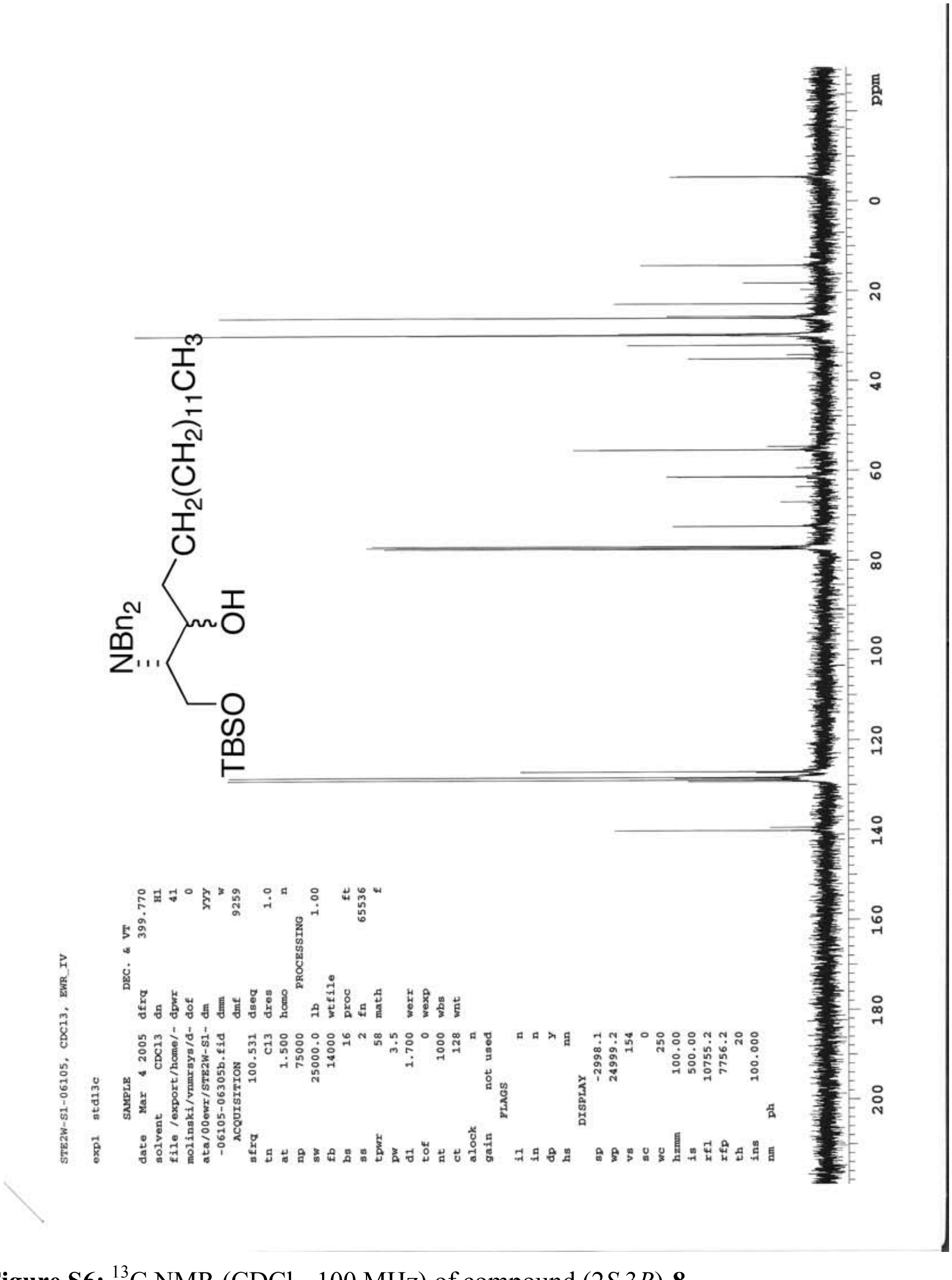

Figure S6: ^{13}C NMR ($CDCl_3$, 100 MHz) of compound (2*S*,3*R*)-**8**

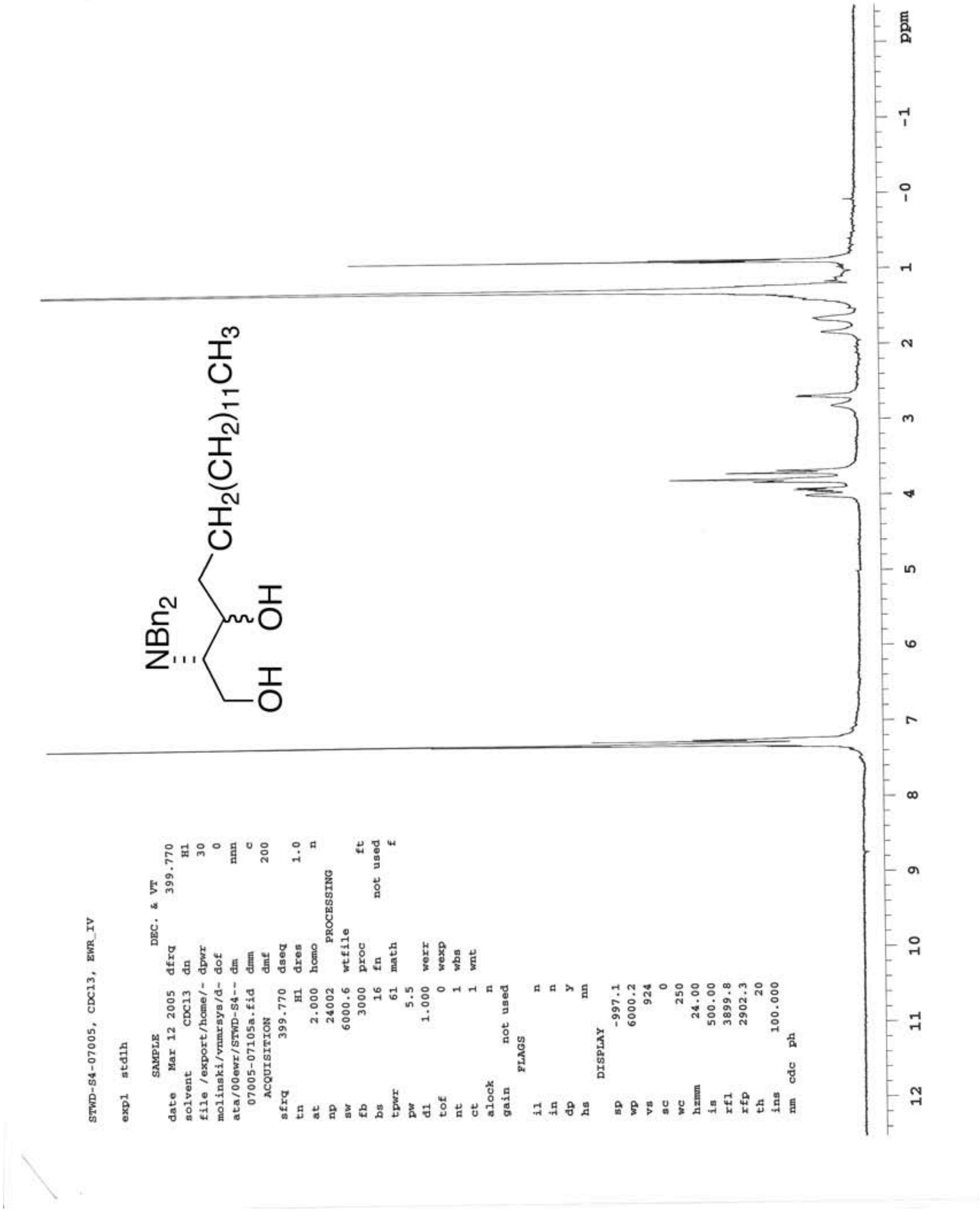

Figure S7: ^{1}H NMR ($CDCl_3$, 400 MHz) of compound (2*S*,3*R*)-**9b**

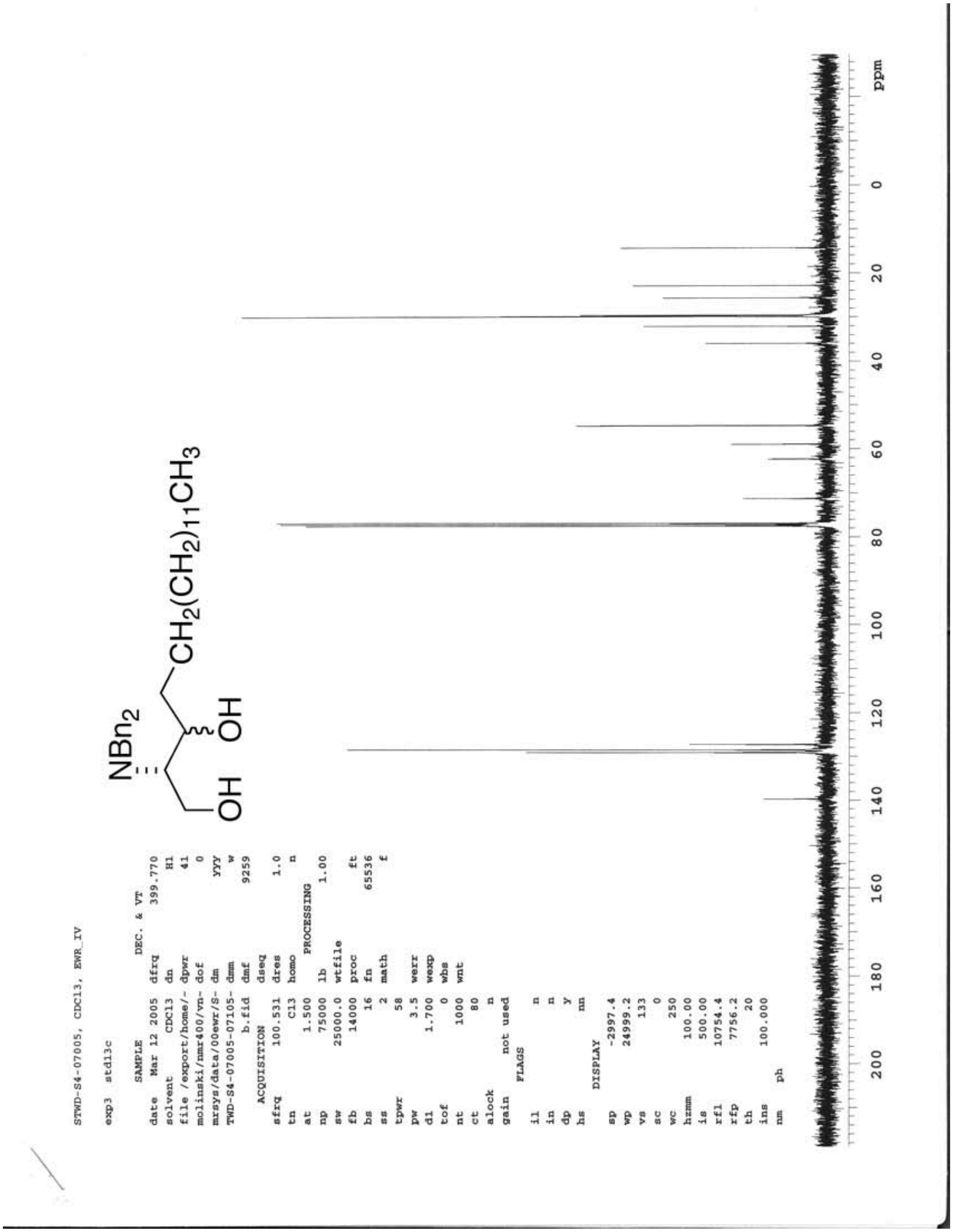

Figure S8: ^{13}C NMR ($CDCl_3$, 100 MHz) of compound (2*S*,3*R*)-**9b**

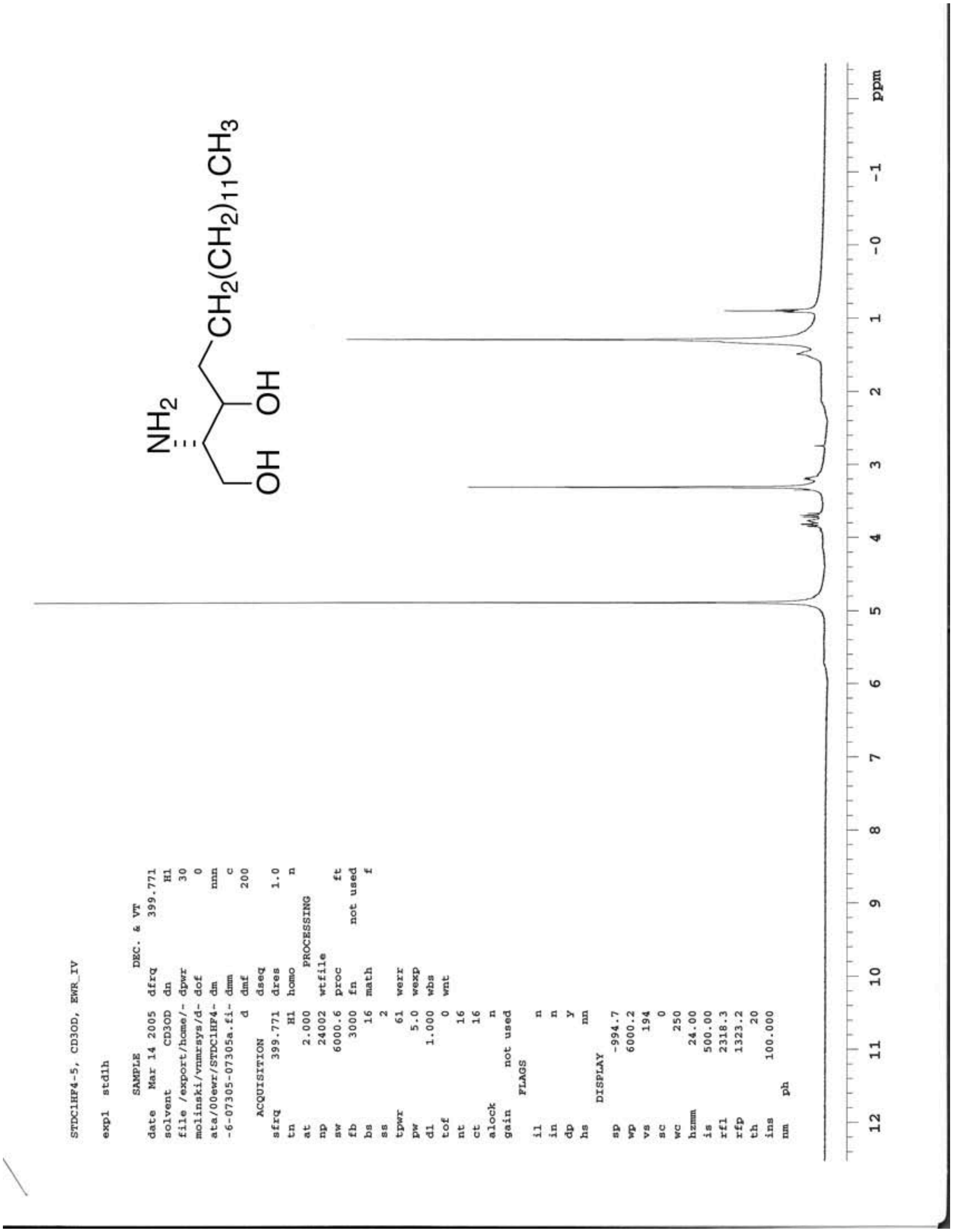

Figure S9: ^{1}H NMR (CD_3OD, 400 MHz) of compound (2*S*,3*R*)-**10** [C_{17}-DHS]

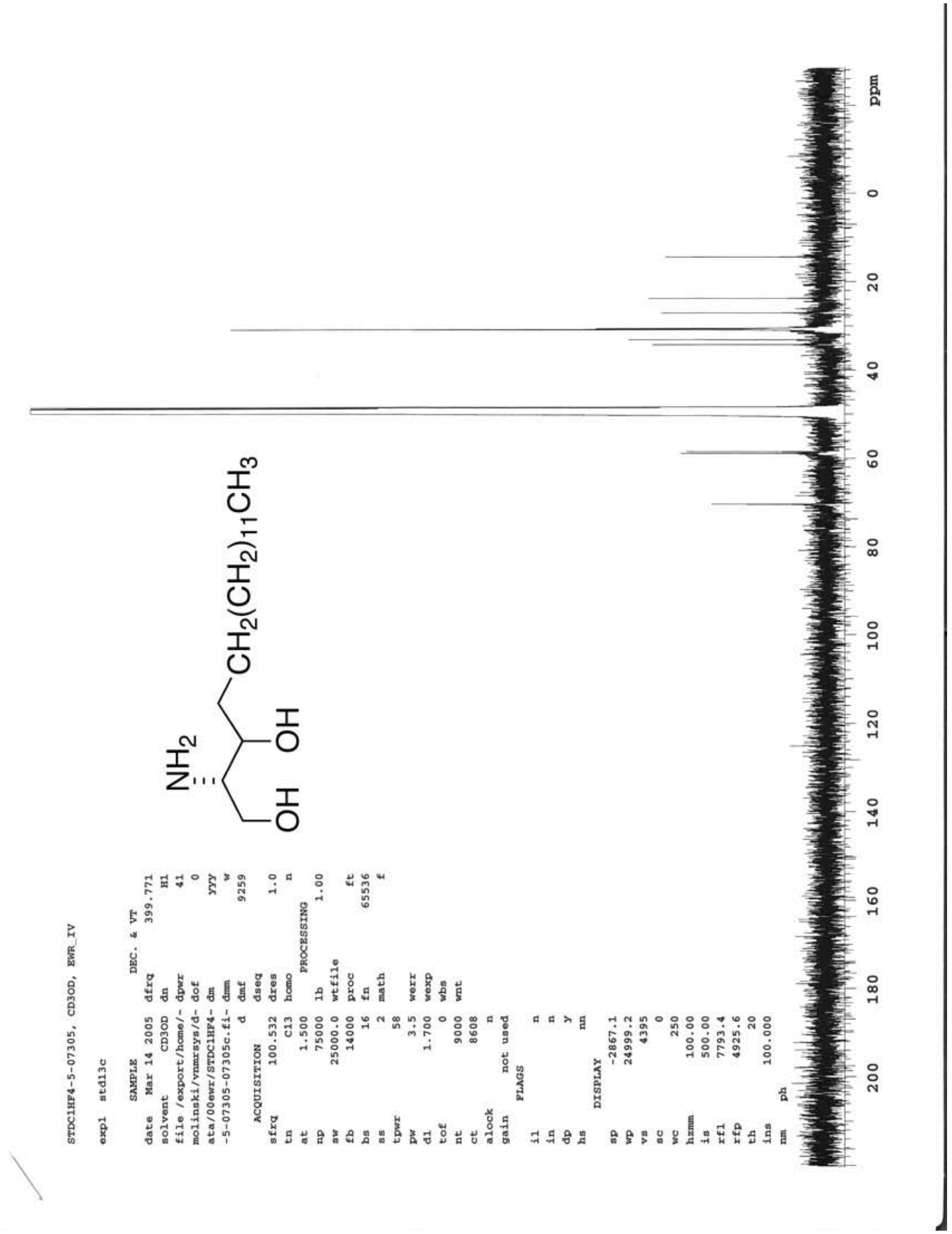

Figure S10: ^{13}C NMR (CD_3OD, 100 MHz) of compound (2*S*,3*R*)-**10** [C_{17}-DHS]

Figure S11. LCMS of Standards PHS+DHS (1:1) (a) 5 µg/mL (b) 50 µg/mL (c) 500 µg/mL (d) PHS+DHS, 500 µg/mL + *C. glabrata* extract. (e, f, g) blanks, MeOH (filtered)

TIC of analytes

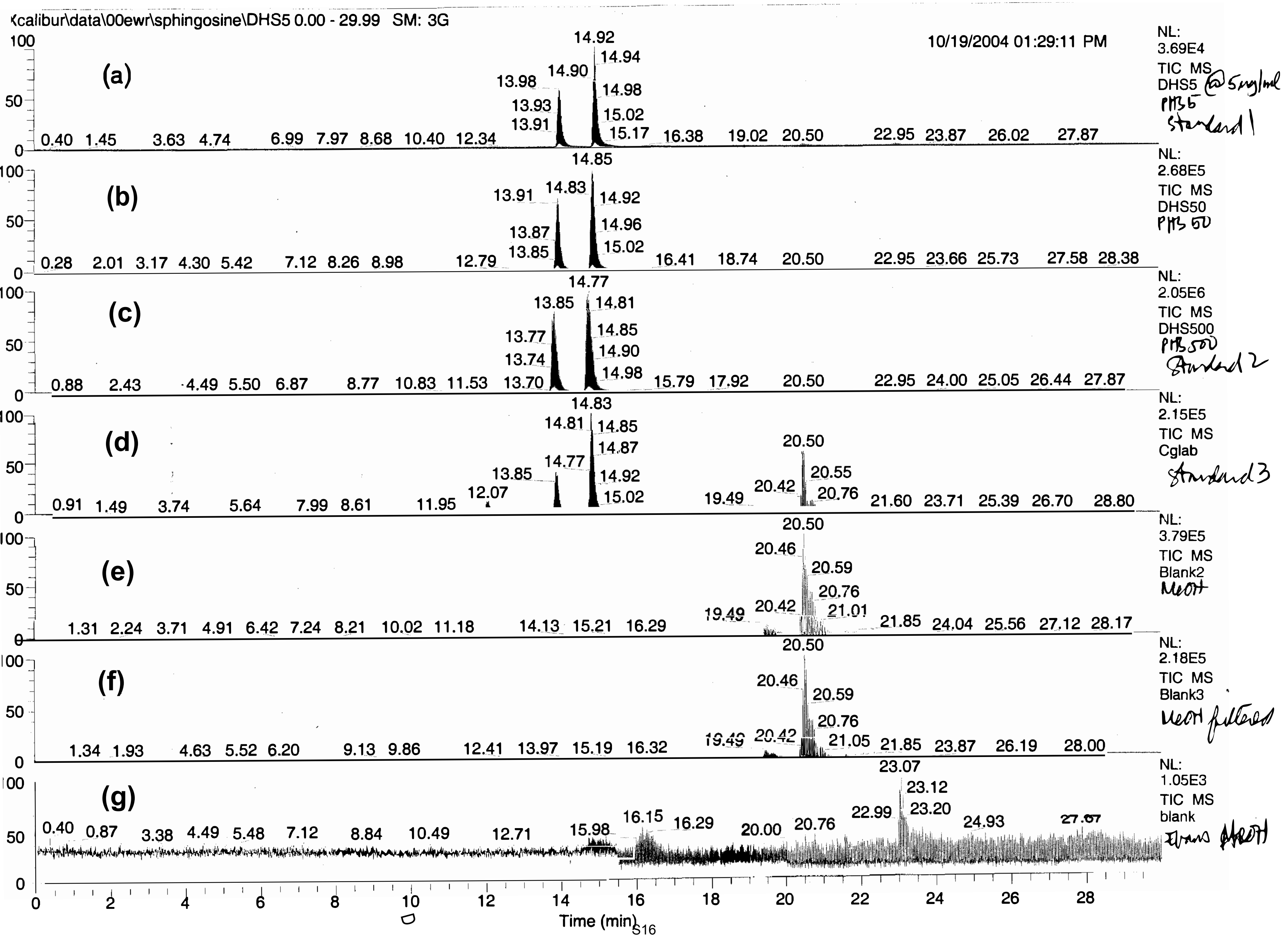

Figure S12; Standard Curves, DHS and PHS.

19-Oct-04 Standards for Quantification: Sphinganine (dihydrosphingosine, DHS) and phytosphingosine (PHS)

	DHS	PHS
5	289682	184559
50	2548293	1724345
500	25279399	18679133

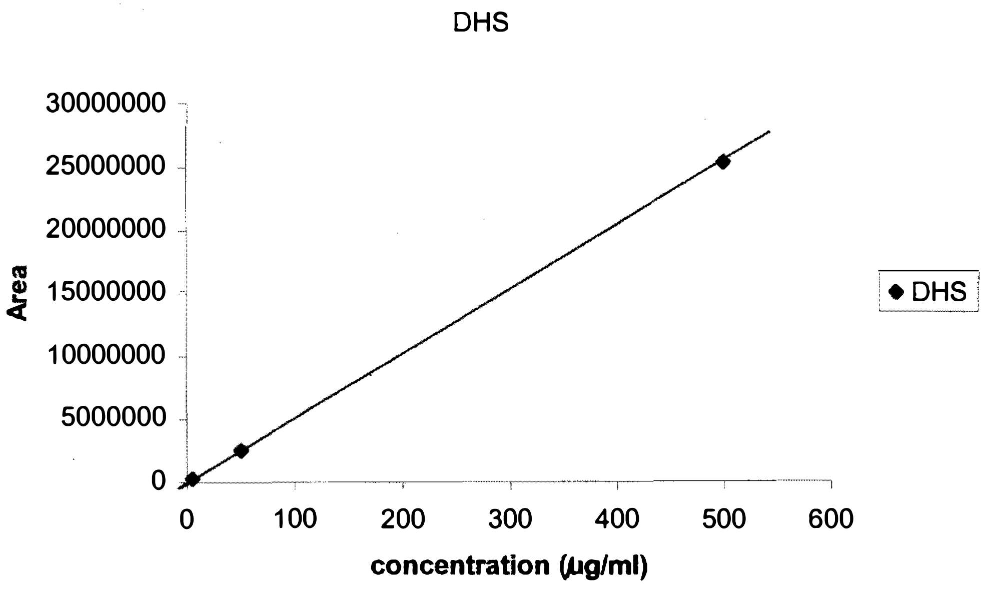

DHS	
intercept	30669.89
slope	50496.15
x	34.00737

Sample:
Area = 1747911
concentration = 34.007 μg/ml

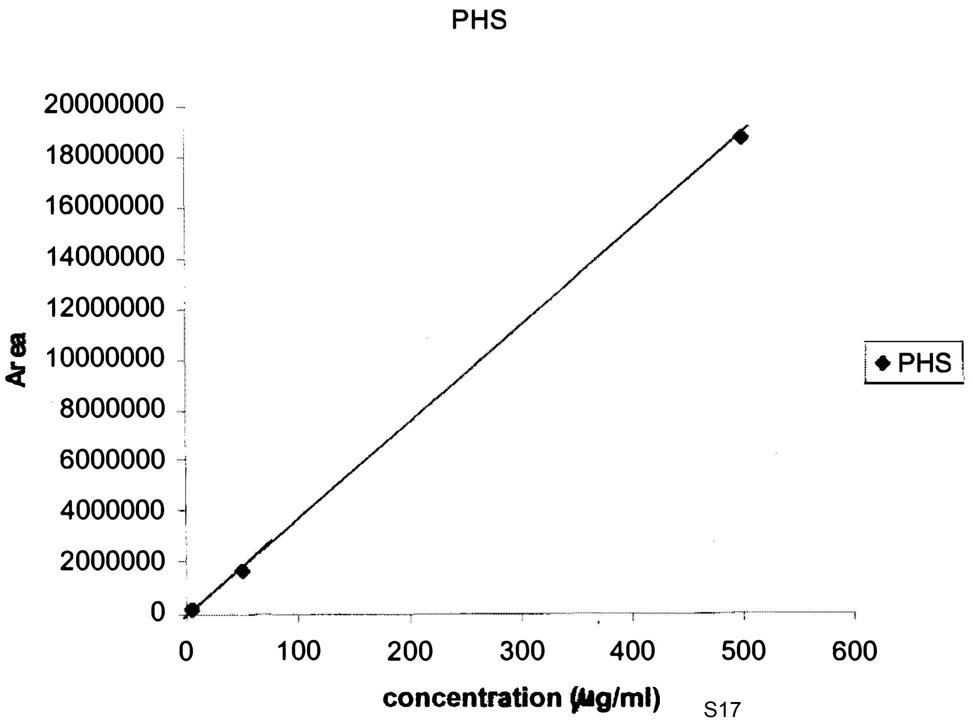

PHS	
intercept	-73024.33
slope	37490.29
x	22.24318

Sample:
Area = 760879
concentration = 22.243 μg/ml
